# Four Novel Dammarane-Type Triterpenoids from Pearl Knots of *Panax ginseng* Meyer cv. Silvatica

**DOI:** 10.3390/molecules24061159

**Published:** 2019-03-23

**Authors:** Zeng Qi, Zhuo Li, Xuewa Guan, Cuizhu Wang, Fang Wang, Pingya Li, Jinping Liu

**Affiliations:** 1 School of Pharmaceutical Sciences, Jilin University, Changchun 130021, China; qizeng95@163.com (Z.Q.); zhuoli198602@gmail.com (Z.L.); wangcz15@mails.jlu.edu.cn (C.W.); 2 Basic Medical College, Jilin University, Changchun 130021, China; guanxw15@mails.jlu.edu.cn (X.G.); wf@jlu.edu.cn (F.W.)

**Keywords:** *Panax ginseng* Meyer cv. Silvatica, new dammarane-type triterpenoids, cigarette smoke, oxidative stress, inflammation

## Abstract

*Panax ginseng* Meyer cv. Silvatica (PGS), which is also known as “Lin-Xia-Shan-Shen” or “Zi-Hai” in China, is grown in forests and mountains by broadcasting the seeds of ginseng and is harvested at the cultivation age of 15–20 years. In this study, four new dammarane-type triterpenoids, ginsengenin-S1 (**1**), ginsengenin-S2 (**2**), ginsenoside-S3 (**3**), ginsenoside-S4 (**4**), along with one known compound were isolated from pearl knots of PGS. Ginsengenin-S2 significantly alleviated oxidative damage when A549 cells were exposed to cigarette smoke (CS) extract. In addition, ginsengenin-S2 could inhibit the CS-induced inflammatory reaction in A549 cells. Protective effects of ginsengenin-S2 against CS-mediated oxidative stress and the inflammatory response in A549 cells may involve the Nrf2 and HDAC2 pathways.

## 1. Introduction

Ginseng, the root of *Panax ginseng* Meyer, a kind of widely used folk medicine in many countries including in China for thousands of years, belongs to the *Araliaceae* family and mainly grows in Korea, China and Japan [1]. *Panax ginseng* Meyer cv. Silvatica (PGS), which is also called “Lin-Xia-Shan-Shen” or “Zi-Hai” in China according to the Chinese Pharmacopoeia, is grown naturally in forests and mountains by broadcasting seeds of ginseng with no human intervention and is harvested at the cultivation age of 15–20 years [2]. In contrast with garden cultivated ginseng (GCG), which is collected after 4–6 years of artificial cultivation in the garden, PGS can imitate the growing environment of wild ginseng [3]. There are obvious verrucous warts on the fibrous roots of PGS, which are called pearl knots (Zhen-zhu-Ge-da) in China. It was reported that the biological nature of pearl knots is the foundation of the seasonal absorbing root of ginseng, in other words, more pearl knots on the PGS indicate longer growth time, while there are scarcely any pearl knots on the GCG [2]. According to the previous report, PGS is of better quality than GCG and exhibited greater anticancer activity than GCG in lung cancer and other cancers [4,5]. However, chemical research of the pearl knots of PGS remains poorly understood. Ginsenosides, the main active principle of *Panax ginseng*, possesses various pharmacological activities including antioxidant [6], immunomodulatory [7], neuroprotective [8], anti-depressive effects [9,10] and renal protective effect [11]. 

Chronic obstructive pulmonary disease (COPD) is “the third killer” in the worldwide public health problem characterized by progressive airflow limitation, progressive lung inflammation and increases in the levels of some inflammatory mediators such as MMP-9, TNF-α and IL-8 [12]. Cigarette smoke (CS) is a proegumenal cause for the development of COPD, as oxidative stress due to CS can drive the severe inflammation of neutrophils and macrophages to the lung and induce apoptosis of epithelial and endothelial cells [13]. It was reported that ginsenoside Rg_1_ could ameliorate CS-induced airway fibrosis and pulmonary epithelial-mesenchymal transition [14,15], while ginsenoside Rb_3_ exerts protective properties against CS extract-induced cell injury in fibroblasts and epithelial cells [16]. 

In this study, four new triterpenoids and one known triterpenoid were isolated from the pearl knots of PGS and we used the CS-stimulated human lung epithelial cells to test their antioxidant activity. Among these five compounds, ginsengenin-S2 exhibited a significant protective effect against CS-induced oxidative stress; then, its protective effects and potential mechanisms against the CS stimulation were preliminarily investigated. 

## 2. Results and Discussion

### 2.1. Structure Elucidation of Compounds ***1***–***5***

**Figure 1:**
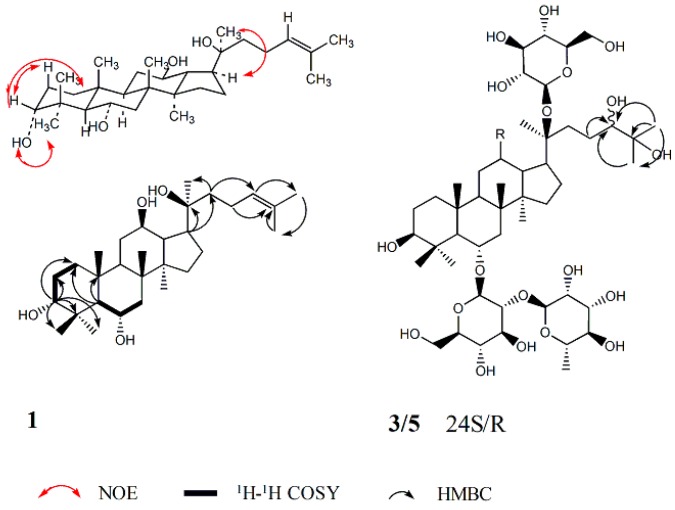
The important NOESY, ^1^H-^1^H COSY and HMBC correlations of **1**; the important HMBC correlations of **3** and **5**. ^1^H-^1^H COSY, ^1^H-^1^H correlation spectroscopy; HMBC, heteronuclear multiple bond correlation; NOESY, nuclear Overhauser effect spectroscopy.

**Figure 2:**
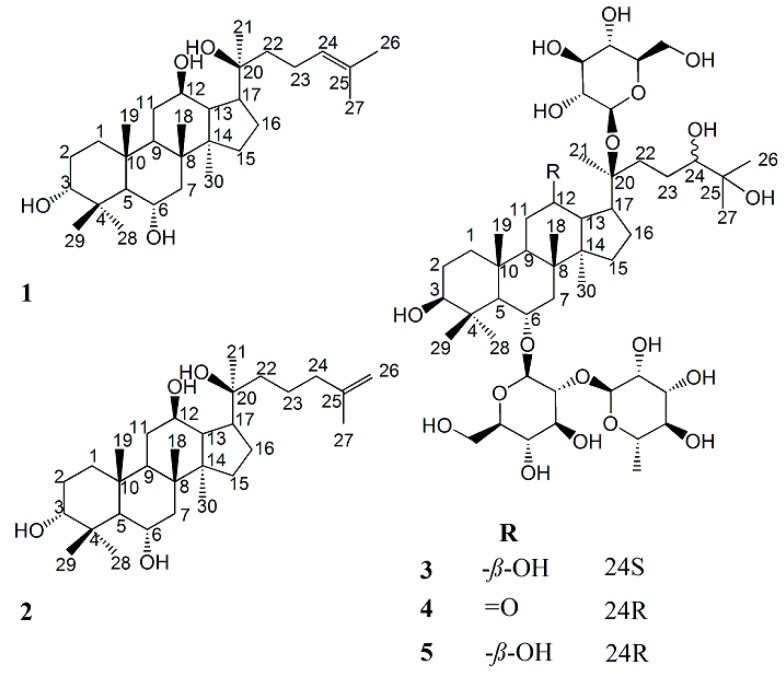
Chemical structures of **1**–**5**: ginsengenin-S1 (**1**); ginsengenin-S2 (**2**); ginsenoside-S3 (**3**); ginsenoside-S4 (**4**); ginsenoside-S5 (**5**).

Compound **1**, white powder: its molecular formula was established as C_30_H_52_O_4_ by HRESIMS. The ^1^H-NMR spectrum of **1** showed eight methyl singlet signals at δ_H_ 0.81, 1.01, 1.11, 1.29, 1.39, 1.62, 1.65, 1.88. The ^13^C-NMR spectrum of **1** showed signals of eight methyl carbons, six quaternary, eight methane and eight methylene among which two carbon signals (δ 126.6 and 131.1) showed the presence of a C=C bond. The chemical shifts of **1** resembled those of protopanaxatriol except the chemical shifts on A ring [17]. The proton and carbon signals of **1** were fully assigned on the basis of 2D-NMR spectra (Table 1). According to the literature, as compared with its 3β-epimers, the C-1~C-5 chemical shifts of 3α-dammarane-type triperpenes are in relatively higher nuclear magnetic fields, while carbon chemical shifts of C-28~C-29 are in relatively lower nuclear magnetic fields. For example, the C-1~C-5 and C-28~C-29 carbon chemical shift values of two pairs of C-3 epimers were shown as follow: [dammar-20*S*,24*S*-epoxy-3α,12β,25-triol: δ_C_ 33.5, 25.4, 76.0, 37.5, 49.5, 28.3, 22.0; dammar-20*R*,24*S*-epoxy-3β,12β,25-triol: δ_C_ 39.0, 27.5, 78.9, 39.0, 55.9, 28.0, 15.4); 3α-ocotillol: δ_C_ 34.9, 26.5, 77.4, 39.1, 56.2, 32.6, 22.8; 3β-ocotillol: δ_C_ 39.4, 28.1, 78.4, 40.4, 61.9, 31.9, 16.5] [18,19]. Carbon chemical shift values of C-1~C-5 and C-28~C-29 on A ring of **1** [δ_C_ 35.0, 26.8, 77.6, 39.3, 56.3, 32.8, 23.1] suggested the existence of α configuration at C-3. NOEs between (i) δ 3.61 (H-3) and 1.29 (H-29) and 2.03 (H-2a), (ii) δ 5.58 (3-OH) and 1.88 (H-28) demonstrated the 3α configuration (Figure 1). Therefore, **1** was elucidated as dammar-24-ene-3α,6α,12β,20*S*-tetraol (ginsengenin-S1, Figure 2).

Compound **2**, white powder: its molecular formula was established as C_30_H_52_O_4_ by HRESIMS. The NMR data of **2** (Table 1) resembled those of **1**. The 3α configuration of **2** was determined by its NOEs the same way as **1**. Compared with **1**, the carbon NMR data of **2** showed the absence of a methyl carbon signal and an additional signal of methylene (δ_C_ 39.3) and a pair of typical signals of a terminal double bond (δ_C_ 110.4 and 146.8) in place of (δ_C_ 126.6 and 131.1), therefore, briefly, the obvious differences between **1** and **2** were the carbon chemical shifts of C-24~C-27. The ^1^H-NMR data of **2** also showed the absence of a methyl hydrogen signal and two additional hydrogen signals of the terminal double bond (δ_H_ 4.80 s, 4.84 s). Thus, **2** was deduced to be dammar-25-ene-3α,6α,12β,20*S*-tetraol (ginsengenin-S2, Figure 2). 

Compounds **3** and **5**, obtained as a white powder. Their molecular formulas were identically established as C_48_H_84_O_20_ by HRESIMS. Acidic hydrolysis of **3** and **5** both produced l-rhamnose and d-glucose, which were detected by GC analysis. Nine methyl singlet signals and a methyl doublet signal were detected in the ^1^H-NMR spectra of both **3** and **5** [**3**: δ_H_ 0.82, 0.89, 1.08, 1.28, 1.46, 1.49, 1.50, 2.00, 1.68 (3H, d, *J* = 6.1 Hz, H-6″); **5**: δ_H_ 0.83, 0.91, 1.11, 1.32, 1.53 (6H, overlapped), 1.55, 2.06, 1.73 (3H, d, *J* = 5.6 Hz, H-6″)], three oxymethine protons [**3**: δ_H_ 3.40 (1H, m, H-3), 3.91 (1H, m, H-12), 3.81 (1H, m, H-24); **5:** δ_H_ 3.43 (1H, m, H-3), 3.87 (1H, m, H-12), 3.74 (1H, m, H-24)], which were attributed to the aglycone moiety, together with three anomeric protons [**3:** δ_H_ 5.12 (1H, m, H-1‴), 5.13 (1H, m, H-1′), 6.35 (1H, brs, H-1″); **5**: δ_H_ 5.17 (1H, m, H-1‴), 5.19 (1H, m, H-1′), 6.43 (1H, brs, H-1″)], assignable to one α-l-rhamnopyranosyl and two β-d-glucopyranosyl units. The ^13^C-NMR data of **3** and **5** showed resemblance with those of ginsenoside Re [20] except the disappearance of C=C bond C-atom signals and the additional appearance of two signals of C-atom [**3**: δ_C_ 79.5 (C-24), 73.1 (C-25); **5**: δ_C_ 80.1 (C-24), 73.1 (C-25)] which connected the hydroxyl group. The following correlations were found in HMBC of **3**: H-26/C-24, C-25, C-27; H-27/C-24, C-25, C-26, which indicated the existence of 2-methylpentane-2,3-diol moiety, and the same phenomenon was observed in **5** (Figure 1). The carbon chemical shifts of glycosyl moiety and the basic parent structure of the aglycone of **5** were consistent with **3** except the side chain (Table 2), which suggested the same nuclear parent of the aglycone and the same type and position of the sugar of **3** and **5**, with only slight differences in the carbon chemical shift values of C-22~C-27 [**3**: δ_C_ 33.2, 26.6, 79.5, 73.1, 27.0, 25.2; **5:** δ_C_ 33.7, 27.2, 80.1, 73.1, 26.9, 25.9]. Therefore, **3** and **5** are a pair of epimers at the 24-position. The carbon chemical shifts of **5** were almost consistent with a saponin isolated from notoginseng through Endophytes biotransformation, of which its structure was identified as 6-*O*-[α-l-rhamnopyranosyl-(1→2)-β-d-glucopyranosyl]-20-*O*-β-d-glucopyranosyl-dammarane-12-one-3,6,20,24,25-hexaol [21], however the absolute configuration of C-24 was not mentioned. By our observation, the carbon chemical shifts of C-22~C-24 and C-27 of **3** were in relatively higher nuclear magnetic fields compared with **5**, and the configuration of the C-24 (δ_C_ 79.5) of **3** was indicated to be 24*S* and the C-24 (δ_C_ 80.1) of **5** was indicated to be 24*R* according to the literatures [tarecilioside A: δ_C_ 80.5 (C-24*R*); notoginsenoside SFt_2_: δ_C_ 80.1 (C-24*R*); notoginsenoside ST-6: δ_C_ 79.4 (C-24*S*); notoginsenoside ST-8: δ_C_ 79.3 (C-24*S*)] [22,23,24,25,26]. Thus, **3** and **5** were inferred as 6-*O*-[α-l-rhamnopyranosyl-(1→2)-β-d-glucopyranosyl]-20-*O*-β-d-glucopyranosyl-dammarane-3β,6α,12β,20*S*,24*S*,25-hexaol (ginsenoside-S3) and 6-*O*-[α-l-rhamnopyranosyl-(1→2)-β-d-glucopyranosyl]-20-*O*-β-d-glucopyranosyl-dammarane-3β,6α,12β,20*S*,24*R*,25-hexaol, respectively (ginsenoside-S3, Figure 2).

Compounds **4** was obtained as a white amorphous powder. The molecular formula was determined as C_48_H_82_O_20_ by HRESIMS. Compared with **5**, the ^1^H-NMR data of **4** showed the appearance of two different chemical shifts of the proton [H-13 (δ_H_ 3.47) and H-17 (δ_H_ 2.96)] and the disappearance of H-12 (δ_H_ 3.87). The ^13^C-NMR spectra of **4** resembled those of **5** with the appearance of a 2-methylpentane-2,3-diol moiety, however, differences in the chemical shift values of C-9, C-11~C-14, C-17 [δ_C_ 54.3, 40.5, 211.6, 56.8, 56.4, 43.5] suggested the emergence of a carbonyl unit at C-12 in **4**. The chemical shifts of **4** were completely assigned using 2D-NMR spectra (Table 2). The configuration of the C-24 (δ_C_ 80.2) of **4** was indicated to be 24*R* according to the C-24 configuration analysis of **3** and **5**, thus the structure of **4** was inferred as 6-*O*-[α-l-rhamnopyranosyl-(1→2)-β-d-glucopyranosyl]-20-*O*-β-d-glucopyranosyl-dammarane-12-one-3β,6α,20*S*,24*R*,25-pentaol (ginsenoside-S4, Figure 2).

### 2.2. Bioactivity Evaluation

#### 2.2.1. Cytotoxicity of Cigarette Smoke Extract (CSE) and Compounds **1**–**5** on A549 Cells

**Figure 3:**
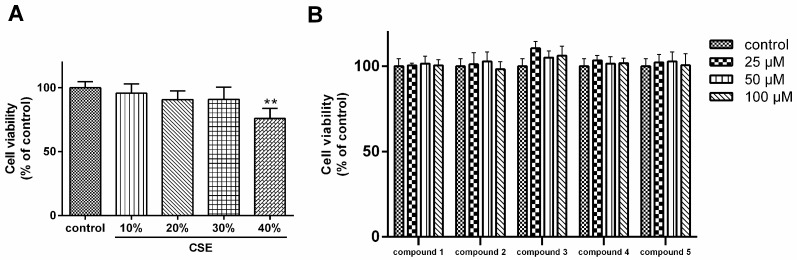
(**A**) Cytotoxicity of cigarette smoke extract (CSE) on A549 cells; (**B**) Effect of compound **1**–**5** on the cell viability of A549 cells. The results represent the mean ± S.D. (*n* = 6). ** *p* < 0.01, compared with control.

The MTT result showed that cell viability of A549 cells was substantially affected by CSE at a concentration≧40% (Figure 3A). Therefore, 30% CSE was used as stimulation in subsequent experiments. As shown in Figure 3B, the cell viability of A549 cells was not affected by **1**–**5**. Then we tested the antioxidant effects of **1**–**5** on CS-stimulated A549 cells. 

#### 2.2.2. Antioxidant Activity of Compounds **1**–**5**

**Figure 4:**
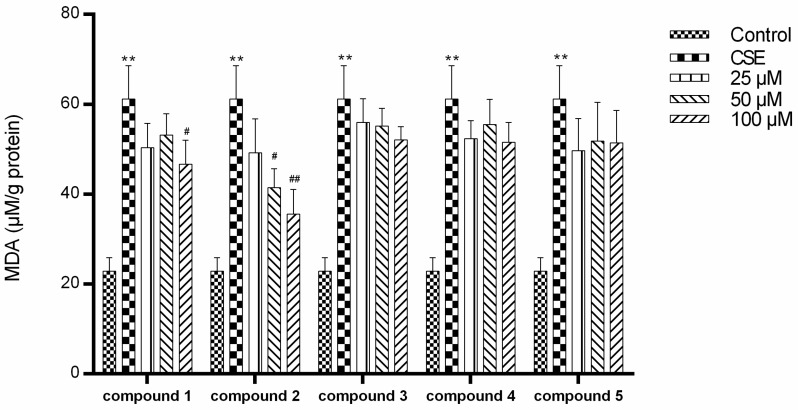
Effect of compounds **1**–**5** on the intracellular malondialdehyde (MDA) level in cigarette smoke (CS)-exposed A549 cells. All data were expressed as mean ± S.D., *n* = 6. ** *p* < 0.01, compared with control; ^#^ *p* < 0.05, ^##^ *p* < 0.01, compared with the CSE group.

As shown in Figure 4, CS exposure significantly increased the intracellular malondialdehyde (MDA) level in human lung epithelial cells. Among these five compounds, ginsengenin-S2 induced a dose-dependent reduction of MDA level.

**Figure 5:**
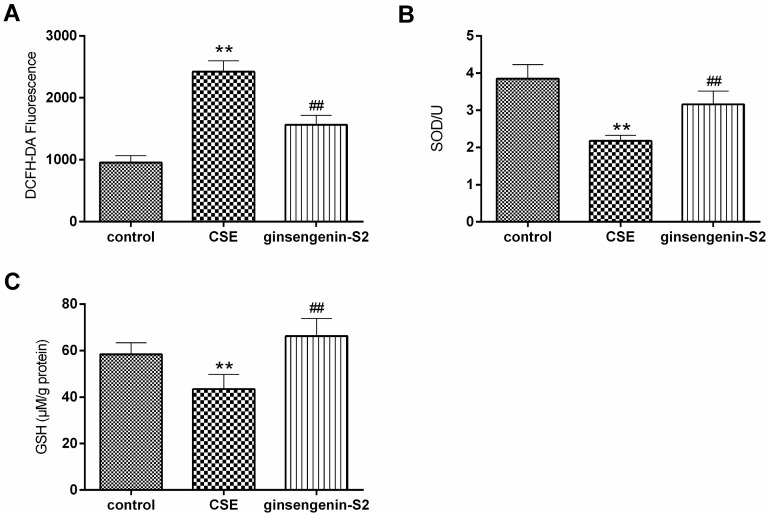
Effects of ginsengenin-S2 (100 μM) on (**A**) the reactive oxygen species (ROS) level, (**B**) intracellular dismutase (SOD) activity and (**C**) glutathione (GSH) level in CS-exposed A549 cells. All data were expressed as mean ± S.D., *n* = 6. ** *p* < 0.01, compared with control; ^##^ *p* < 0.01, compared with the CSE group.

Tobacco smoke is involved in the increased synthesis of reactive oxygen species (ROS) [27]. In our study, CS exposure induced significantly higher ROS level in A549 cells than the control group (Figure 5A). Interestingly, ginsengenin-S2 down-regulated the ROS level in CS exposed-A549 cells. Then, we investigated the role of ginsengenin-S2 on the activities of two first line defense antioxidants-superoxide dismutase (SOD) and glutathione (GSH) in CS-exposed human lung epithelial cells. As shown in Figure 5B,C, the CS challenge caused the decrease of intracellular GSH content and SOD activity (*p* < 0.01), with a parallel of increased MDA level which demonstrates that the CS challenge induced oxidative stress in the in vitro cell model. Further, we observed that pretreatment with ginsengenin-S2 could attenuate CS-induced oxidative stress by clearly up-regulating the content of SOD and GSH to a relatively basal level. 

#### 2.2.3. Effect of Ginsengenin-S2 on CS-Induced IL-8 Levels In Vitro

**Figure 6:**
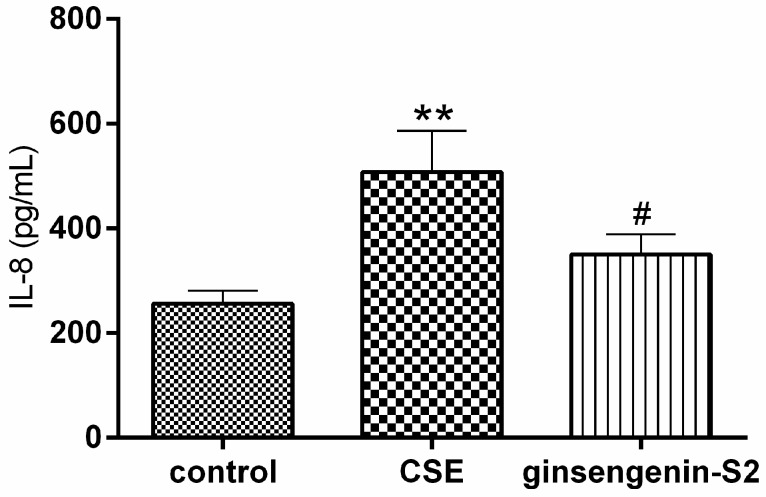
Anti-inflammatory effect of ginsengenin-S2 (100 μM) on the inflammatory cytokine interleukin-8 (IL-8) in CS-exposed A549 cells. All data were expressed as mean ± S.D., *n* = 4. ** *p* < 0.01, compared with control; ^#^ *p* < 0.05, compared with the CSE group.

CS is also a leading risk factor for the development of an inflammatory condition characterized by the release of proinflammatory mediators such as interleukin-8 (IL-8). We then investigated whether ginsengenin-S2 inhibited the release of chemokine IL-8 from CS-stimulated lung epithelial cells. As shown in Figure 6, IL-8 production markedly rose in the cell treated with CS (*p* < 0.01), however pretreatment with ginsengenin-S2 at 100 μM resulted in a decreased IL-8 level (*p* < 0.05).

#### 2.2.4. Effect of Ginsengenin-S2 on CS-Mediated Protein Expression of Nrf2 and HDAC2 In Vitro

**Figure 7:**
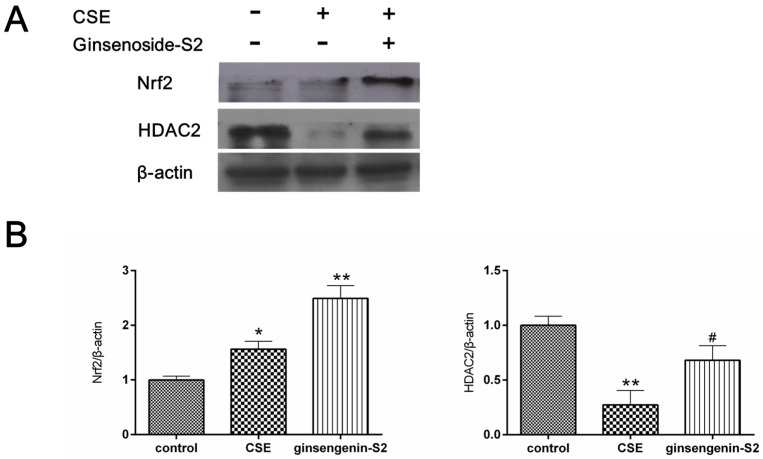
(**A**) Effect of ginsengenin-S2 (100 μM) on the protein expression of nuclear-related factor 2 (Nrf2) and histone deacetylase 2 (HDAC2) in the CS-exposed A549 cells was examined by western blot analysis; (**B**) quantitative analyses of Nrf2 in each group. Data are expressed as mean ± SD (*n* = 3). * *p* < 0.05, ** *p* < 0.01 as compared to the control group; ^#^ *p* < 0.05 as compared to the CSE treated group.

To explore the underlying mechanism of the antioxidant activity and anti-inflammatory property of ginsengenin-S2, we examined the effect of ginsengenin-S2 on the protein expression of nuclear-related factor 2 (Nrf2) and histone deacetylase 2 (HDAC2) in CS-exposed A549 cells. In our study, we observed that the expression level of Nrf2 increased and the level of HDAC2 decreased after the CS challenge (*p* < 0.05). Interestingly, treatment with ginsengenin-S2 significantly activated the expression of Nrf2 (*p* < 0.01) as compared to the control group (Figure 7), which suggested that Nrf2 may participate in the meditative effect of ginsengenin-S2 in CS-induced oxidative stress. In addition, ginsengenin-S2 up-regulated the expression of HDAC2 in CS-exposed lung epithelial cells (*p* < 0.05).

Oxidative stress is involved in COPD pathogenesis and could cause the enrichment of ROS and even leads to organ tissue damage [28,29]. CS is one of the major risk contributors to COPD which could increase ROS production. As an important role in anti-oxidation, GSH could help reduce the production of lipid peroxide and prevent cell damage [30]. SOD is an enzyme which could scavenge superoxide radicals. It is reported that SOD and GSH-associated enzymes in the lungs of COPD patients are significantly affected by CS [31]. MDA, an important marker for monitoring the process of membrane lipid peroxidation and damage degrees, appears to have a different level between healthy smokers and COPD patients [32]. The current data suggested that ginsengenin-S2, a novel sapogenin, noticeably attenuated CS-induced oxidative damage and inflammatory action via up-regulating the content of intracellular SOD and MDA and inhibiting the level of MDA and IL-8 release. Nrf2, an important antioxidant transcription factor against oxidative stress, is released from the Keap1-Nrf2 complex and translocates from the cytoplasm when cells are exposed to oxidative stress. Increasing studies show that Nrf2 mediates the protective effects of natural products on CS exposure caused oxidative stress and inflammatory responses [16,33]. CS-induced lung inflammation involves the reduction of HDAC2 which is related to steroid resistance in patients with COPD who smoke cigarettes [34]. In this study, we found that ginsengenin-S2 activated the Nrf2 and enhanced the HDAC2 activity, suggesting that the protective effect of ginsengenin-S2 may involve the Nrf2 and HDAC2 pathways. To better utilize the medicinal resource of *Panax ginseng* Meyer cv. Silvatica, it is necessary to study the protective effect of its root extract against the cigarette smoke-induced COPD or airway fibrosis in vivo in the future.

## 3. Materials and Methods

### 3.1. Plant Materials

PGS was provided by the Guandong Green Ecology Ginseng & Pantotrichum Co., Ltd (Fusong, Jilin, China) in 2016 and was identified by Professor Pingya Li of Jilin University. The voucher specimen (No. PGS161220) was deposited at the National and Local United Engineering R&D Center of Ginseng Innovative Drugs, China. 

### 3.2. Apparatus and Chemicals

HRESIMS was performed on Waters Xevo G2-XS QTOF mass spectrometer (Waters Co., Milford, MA, USA). Gas chromatography (GC) was carried out on an Agilent 5979 GC-MS (Agilent, J&W Scientific, Folsom, CA, USA). IR spectra were taken on WGH-30/6 drug-beam infrared spectrophotometer (Gangdong Technology Co. Ltd., Tianjin, China). The NMR spectra were measured on Bruker AV-600 spectrometer (Bruker Co., Karlsruhe, Germany) with tetramethylsilane (Sigma-Aldrich Co., St. Louise, MO, USA) as the internal standard. Column chromatography (CC) was performed with silica gel (200-300 mesh; Anhui Liangchen Silicon Material Co. Ltd., Huoshan, China). Thin-layer chromatography (TLC) was conducted on silica gel GF254 plates (Anhui Liangchen Silicon Material Co. Ltd., Huoshan, China). Preparative HPLC was carried out using a Shodex R1-201H Refractive Index Detector (Kexiao Chemical Equipment Co., Hangzhou, China) and YMC Prep C18 Column (5 μm, 20 mm × 10 mm; YMC Co., Ltd., Kyoto, Japan), 1525 Binary HPLC pump (Waters Co., Milford, MA, USA.). Methanol (MeOH) and acetonitrile (ACN) (HPLC grade, Janssens Pharmaceuticalaan Co., Fair Lawn, NJ, USA), ultrapure water (Wahaha Co., Ltd, Hangzhou, China) and other solvents used were of analytical grade (Tianjin Fine Chemical Works, Tianjin, China). 

### 3.3. Extraction and Isolation

The pearl knots of PGS (1 kg) were completely extracted with 95% ethanol (EtOH) and the ethanolic extracts were concentrated under vacuum. The ethanolic extracts (82 g) were subjected to macro-reticular absorption resin (D101) and began with H_2_O and then were eluted with 95% EtOH. The EtOH fraction (65 g) was then subjected to silica gel CC (200–300 mesh) and was eluted with CH_2_Cl_2_-MeOH (gradient 100:1 to 1:100) to give 41 fractions (Fr 1–41). Fr 7 (1.3 g) was further separated by silica gel CC [petroleum ether (PE)-ethyl acetate (EtOAc), gradient 1:1 to 1:4] to afford three sub-fractions (Fr 7a–7c), Fr 7c (0.6 g) was followed by preparative RP-HPLC (C18 Column, 5 μm, 20 mm × 10 mm) with ACN-H_2_O (44:56, 4 mL/min) as a mobile phase to afford compounds **6** (30 mg, 24 min), **1** (7 mg, 35 min) and **2** (4 mg, 39 min). Fr 24 (1.8 g) was further separated by silica gel CC (EtOAc-EtOH, 10:1 to 2:1) to obtain four sub-fractions (Fr 24a–24d), compound **7** (0.3 g) was recrystallized from Fr 24a (0.5 g) in MeOH, Fr 24b (0.4 g) was followed by preparative RP-HPLC (C18 Column, 5 μm, 20 mm × 10 mm) with ACN-H_2_O (20:80, 3 mL/min) as a mobile phase to afford compounds **3** (17 mg, 11 min), **5** (20 mg, 19 min) and **4** (2 mg, 22 min). 

### 3.4. Spectroscopic Data

#### 3.4.1. Ginsengenin-S1 (**1**)

Dammar-24-ene-3α,6α,12β,20*S*-tetraol: white amorphous powder; IR *ν*_max_ 3394, 2960, 1632, 1455, 1383, 1032, 609 cm^−1^; Libermann-Burchard reaction was positive, suggesting the existence of triterpenoid structure; ^1^H and ^13^C-NMR: see Table 1; HRESIMS *m/z* 521.3880 [M + HCOO]^−^ (calculated for C_31_H_53_O_6_, 521.3842); chemical spectra see in the Supplementary Materials.

#### 3.4.2. Ginsengenin-S2 (**2**)

Dammar-25-ene-3α,6α,2β,20*S*-tetraol: white amorphous powder; IR *ν*_max_ 3328, 2964, 1653, 1461, 1386, 1111, 1028, 649 cm^−1^; Libermann-Burchard reaction was positive, suggesting the existence of triterpenoid structure; ^1^H and ^13^C-NMR: see Table 1; HRESIMS *m/z* 521.3844[M + HCOO]^−^ (calculated for C_31_H_53_O_6_, 521.3842); chemical spectra see in the Supplementary Materials.

#### 3.4.3. Ginsenoside-S3 (**3**)

6-*O*-[α-l-rhamnopyranosyl-(1→2)-β-d-glucopyranosyl]-20-*O*-β-d-glucopyranosyl-dammarane-3β,6α,12β,20*S*,24*S*,25-hexaol: white amorphous powder; IR *ν*_max_ 3406, 2944, 1659, 1371, 1032, 597 cm^−1^; Libermann-Burchard reaction was positive, suggesting the existence of triterpenoid structure; Molish reactions were positive, suggesting the existence of glucoside; ^1^H and ^13^C-NMR: see Table 2; HRESIMS *m/z* 1025.5554 [M + HCOO]^−^ calculated for C_49_H_85_O_22_, 1025.5532); chemical spectra see in the Supplementary Materials.

#### 3.4.4. Ginsenoside-S4 (**4**)

6-*O*-[α-l-rhamnopyranosyl-(1→2)-β-d-glucopyranosyl]-20-*O*-β-d-glucopyranosyl-dammarane-12-one-3β,6α,20*S*,24*R*,25-pentaol: white amorphous powder; IR *ν*_max_ 3412, 2944, 1626, 1068,580 cm^−1^; Libermann-Burchard reaction was positive, suggesting the existence of triterpenoid structure; Molish reactions were positive, suggesting the existence of glucoside; ^1^H and ^13^C-NMR: see Table 2; HRESIMS *m/z* 1023.5424 [M + HCOO]^−^ (calculated for C_49_H_83_O_22_, 1023.5376); chemical spectra see in the Supplementary Materials.

### 3.5. Bioassay

#### 3.5.1. Preparation of Cigarette Smoke Extract

Xiongshi cigarette (China Tobacoo Zhejiang Industrial Co., Ltd, Hangzhou, China; each cigarette contained 0.7 mg of nicotine, 8 mg of tar and 10 mg of carbon monoxide) were used to prepare the cigarette smoke extract (CSE) according to the literature [35]. In brief, one Xiongshi cigarette was bubbled through 25 mL of culture medium to generate 100% concentration CSE, then the solution was filtered through the 0.22 μm pore filter. The CSE (pH 7.2–7.4) was freshly prepared for each experiment and was then diluted to the desired concentration and used within 30 min. 

#### 3.5.2. Cell Viability Assay

The triterpenoids were dissolved in DMSO as stock solutions and diluted with DMEM to the needed concentrations. Final DMSO concentration in the sample was less than 0.1%. Cell culture and MTT assay were done as we described previously to evaluate 24 h-incubation of triterpenoids or the 18 h-incubation of CSE on the cell viability of A549 cells [6]. 

#### 3.5.3. Drug Treatment

A549 cells were cultured in 6-well plates at a density of 5 × 10^5^ cells per 1 mL and were pretreated with triterpenoids for 6 h. After that, the cells were treated with both CSE and triterpenoids for another 18 h of incubation. 

#### 3.5.4. Determination of Oxidative Stress

After 18 h of stimulation of CSE, the levels of intracellular MDA, GSH and SOD in the lysates were measured using commercial MDA, GSH and SOD kits (Jiancheng Bioengineering Institute, Nanjing, China) according to the manufacturer’s protocol.

#### 3.5.5. Reactive Oxygen Species (ROS) Assay

A549 cells were cultured in 96-well plates (5 × 10^3^ cells per well). Drug treatment and CS stimulation were carried out as described in Section 3.5.3 and Section 3.5.4. The cells were washed with warm PBS and the ROS detection was performed with DCFH-DA probe based ROS Assay Kit (Beyotime Biotechnology, Shanghai, China) at 37 °C. The ROS level was expressed as the fluorescence intensity.

#### 3.5.6. Enzyme-Linked Immunosorbent Assay

After 18 h of stimulation of CSE, IL-8 content in the cell culture supernatant was measured using IL-8 ELISA kit (R&D Systems, Minneapolis, MN, USA) according to the manufacturer’s protocol.

#### 3.5.7. Western Blotting

Western blot assay was performed as previous described [6] to study the effects of ginsengenin-S2 on the protein expression of HDAC2 and Nrf2 in CSE-stimulated A549 cells. Anti-HDAC2 (#ab32117), anti-Nrf2 (#ab137550) and anti-β-actin (#ab137550) were from Abcam Company (Cambridge, UK). The HDAC2 and Nrf2 band intensities were adjusted with reference to β-actin control.

#### 3.5.8. Statistical Analysis

Data analysis was performed using GraphPad Prism 6.0 software (GraphPad Software, San Diego, USA). All the data are presented as mean ± S.D. and a two tailed test or a one-way analysis of variance (ANOVA) was used to determine the statistical significance, and *p*-value <0.05 was considered as significant.

## 4. Conclusions

In our study, four new dammarane-type triterpenoids, elucidated as ginsengenin-S1 (**1**), ginsengenin-S2 (**2**), ginsenoside-S3 (**3**) and ginsenoside-S4 (**4**) were isolated from pearl knots of PGS and their antioxidant activities were investigated using CS-stimulated A549 cells. Among these compounds, ginsengenin-S2 exhibited protective activity against CS-induced oxidative damage and ameliorated inflammatory reaction. Further, its protective effect against CS may involve the Nrf2 and HDAC2 pathways. This study revealed the evidence that an active compound from the root of *Panax ginseng* Meyer cv. Silvatica—ginsengenin-S2 is beneficial to reduce injury from cigarette smoke in vitro and provides some theoretical basis for the development and utilization of the resource *Panax ginseng* Meyer cv. Silvatica. It reminded us that it is worthwhile to study the in vivo protective effect of the root of *Panax ginseng* Meyer cv. Silvatica against cigarette smoke using animal models.

## Figures and Tables

**Table 1:** ^1^H and ^13^C-NMR (600 MHz, 150 MHz in C_5_D_5_N) data for **1** and **2**.

Position	1		2		Position	1		2	
	δ_C_	δ_H_	δ_C_	δ_H_		δ_C_	δ_H_	δ_C_	δ_H_
1	35	1.85 (1H, m)	35.1	1.88 (1H, m)	17	55	2.27 (1H, m)	55.1	2.30 (1H, m)
		1.48 (1H, m)		1.49 (1H, m)	18	17.7	1.11 (3H, s)	17.7	1.20 (3H, s)
2	26.8	2.03 (1H, m)	26.9	2.05 (1H, m)	19	18.4	1.01 (3H, s)	18.5	1.05 (3H, s)
		1.80 (1H, m)		1.80 (1H, m)	20	73.3		73.4	
3	77.6	3.61 (1H, d, 10)	77.7	3.63 (1H, br s)	21	27.3	1.39 (3H, s)	27.6	1.39 (3H, s)
4	39.3		39.4		22	36.1	2.01 (1H, m)	35.8	1.96 (1H, m)
5	56.3	1.89 (1H, m)	56.4	1.93 (1H, m)			1.67 (1H, m)		1.62 (1H, m)
6	67.6	4.33 (1H, m)	67.7	4.38 (1H, m)	23	23.3	2.58 (1H, m)	22.8	2.03 (1H, m)
7	47.6	1.94 (1H, m)	47.7	1.97 (1H, m)			2.25 (1H, m)		1.67 (1H, m)
		1.88 (1H, m)		1.79 (1H, m)	24	126.6	5.30 (1H, t, 6.3)	39.3	2.08 (1H, m)
8	41.4		41.4		25	131.1		146.8	
9	50.3	1.75 (1H, m)	50.4	1.78 (1H, m)	26	26.15	1.65 (3H, s)	110.4	4.84 (1H, s)
10	39.5		39.6						4.80 (1H, s)
11	32.4	2.19 (1H, m)	32.6	2.25 (1H, m)	27	18	1.62 (3H, s)	23	1.71 (3H, s)
		1.57 (1H, m)		1.63 (1H, m)	28	32.8	1.88 (3H, s)	32.9	1.91 (3H, s)
12	71.4	3.86 (1H, m)	71.4	3.89 (1H, m)	29	23.1	1.29 (3H, s)	23.2	1.32 (3H, s)
13	48.5	2.02 (1H, m)	48.6	2.07 (1H, m)	30	17.3	0.81 (3H, s)	17.3	0.83 (3H, s)
14	52		52.1		3-OH		5.58 (1H, br s)		5.59 (1H, d, 4.0)
15	31.6	1.58 (1H, m)	31.7	1.62 (1H, m)	6-OH		5.15 (1H, br s)		5.17 (1H, d, 6.9)
		1.00 (1H, m)		1.03 (1H, m)	12-OH		7.22 (1H, s)		7.23 (1H, s)
16	27.1	1.81 (1H, m)	27.2	1.84 (1H, m)	20-OH		6.96 (1H, s)		6.96 (1H, s)
		1.35 (1H, m)		1.37 (1H, m)					

Delta is ppm of chemical shifts which are reported in parts per million (δ), and coupling constants (*J*) are expressed in Hertz.

**Table 2:** ^1^H and ^13^C-NMR (600 MHz, 150 MHz in C_5_D_5_N) data for **3**–**5**.

Position	3		4		5	
	δ_C_	δ_H_	δ_C_	δ_H_	δ_C_	δ_H_
1	39.4	1.59 (1H, m)	39.4	1.39 (1H, m)	39.5	1.62 (1H, m)
		0.86 (1H, m)		0.87 (1H, m)		0.89 (1H, m)
2	27.6	1.76 (2H, m)	27.9	1.77 (1H, m)	27.8	1.83 (1H, m)
				1.81 (1H, m)		1.77 (1H, m)
3	78.5	3.40 (1H, m)	78.5	3.46 (1H, m)	78.6	3.43 (1H, m)
4	39.9		39.7		40.1	
5	60.8	1.30 (1H, m)	60.8	1.39 (1H, m)	61.0	1.34 (1H, m)
6	74.5	4.58 (1H, m)	74.5	4.75 (1H, m)	74.7	4.62 (1H, m)
7	45.9	2.12 (1H, m)	45.8	2.34 (1H, m)	46.0	2.19 (1H, m)
		1.86 (1H, m)		1.92 (1H, m)		1.91 (1H, m)
8	41.1		42.1		41.3	
9	49.5	1.42 (1H, m)	54.3	1.84 (1H, m)	49.6	1.46 (1H, m)
10	39.6		40.0		39.8	
11	30.8	1.99 (1H, m)	40.5	2.27 (2H, m)	31.1	2.01 (1H, m)
		1.38 (1H, m)				1.38 (1H, m)
12	70.6	3.91 (1H, m)	211.6		70.7	3.87 (1H, m)
13	48.6	1.97 (1H, m)	56.8	3.47 (1H, m)	48.9	1.96 (1H, m)
14	51.5		56.4		51.7	
15	30.9	1.41 (1H, m)	32.3	1.80 (1H, m)	31.0	1.45 (1H, m)
		0.74 (1H, m)		0.96 (1H, m)		0.81 (1H, m)
16	26.7	1.63 (1H, m)	27.3	2.44 (1H, m)	26.9	1.67 (1H, m)
		1.22 (1H, m)		1.99 (1H, m)		1.24 (1H, m)
17	52.8	2.32 (1H, m)	43.5	2.96 (1H, m)	53.0	2.30 (1H, m)
18	17.2	1.08 (3H, s)	17.5	1.49 (3H, s)	17.3	1.11 (3H, s)
19	17.5	0.89 (3H, s)	17.8	0.98 (3H, s)	17.7	0.91 (3H, s)
20	83.9		82.2		83.6	
21	22.8	1.49 (3H, s)	22.8	1.53 (3H, s)	23.0	1.53 (3H, overlapped)
22	33.2	2.35 (2H, m)	38.5	2.57 (1H, m)	33.7	2.90 (1H, m)
				2.07 (1H, m)		1.85 (1H, m)
23	26.6	2.41 (1H, m)	25.2	2.18 (1H, m)	27.2	2.17 (1H, m)
		1.78 (1H, m)		1.85 (1H, m)		2.00 (1H, m)
24	79.5	3.81 (1H, m)	80.2	3.77 (1H, m)	80.1	3.74 (1H, m)
25	73.1		73.3		73.1	
26	27.0	1.50 (3H, s)	26.4	1.55 (3H, s)	26.9	1.55 (3H, s)
27	25.2	1.46 (3H, s)	26.4	1.54 (3H, s)	25.9	1.53 (3H, overlapped)
28	32.1	2.00 (3H, s)	32.5	2.10 (3H, s)	32.3	2.06 (3H, s)
29	17.6	1.28 (3H, s)	17.8	1.35 (3H, s)	17.8	1.32 (3H, s)
30	17.1	0.82 (3H, s)	17.2	0.85 (3H, s)	17.2	0.83 (3H, s)
6-glc-1′	101.8	5.13 (1H, m)	102.1	5.25 (1H, d, 6.3)	102.0	5.19 (1H, m)
2′	78.7	4.14 (1H, m)	78.7	3.97 (1H, m)	78.7	4.18 (1H, m)
3′	78.6	4.26 (1H, m)	78.4	4.36 (1H, m)	79.0	4.31 (1H, m)
4′	72.5	4.12 (1H, m)	72.9	4.21 (1H, m)	72.7	4.16 (1H, m)
5′	78.3	3.88 (1H, m)	79.4	4.20 (1H, m)	78.7	3.92 (1H, m)
6′	63.0	4.42 (1H, m)	63.4	4.51 (1H, m)	63.3	4.52 (1H, m)
		4.16 (1H, m)		4.36 (1H, m)		4.17 (1H, m)
2′-rham-1″	101.9	6.35 (1H, brs)	102.3	6.48 (1H, d, 6.3)	102.0	6.43 (1H, brs)
2″	72.3	4.70 (1H, m)	72.8	4.80 (1H, m)	72.5	4.76 (1H, brs)
3″	72.2	4.57 (1H, m)	72.6	4.67 (1H, m)	72.4	4.64 (1H, m)
4″	74.0	4.25 (1H, m)	74.5	4.30 (1H, m)	74.3	4.30 (1H, m)
5″	69.5	4.83 (1H, td, 9.2, 6.3)	69.8	4.94 (1H, m)	69.6	4.89 (1H, m)
6″	18.7	1.68 (3H, d, 6.1)	19.1	1.76 (3H, d, 5.9)	18.9	1.73 (3H, d, 5.6)
20-glc-1‴	98.3	5.12 (1H, m)	98.7	5.10 (1H, m)	98.5	5.17 (1H, m)
2‴	75.2	3.89 (1H, m)	76.0	3.96 (1H, m)	75.6	3.95 (1H, m)
3‴	79.3	4.25 (1H, m)	79.8	4.36 (1H, m)	79.5	4.30 (1H, m)
4‴	71.6	3.98 (1H, t, 9.1)	72.2	4.14 (1H, m)	71.7	3.98 (1H, m)
5‴	78.1	3.88 (1H, m)	78.8	3.97 (1H, m)	78.5	3.89 (1H, m)
6‴	62.9	4.42 (1H, m)	63.4	4.51 (1H, m)	63.2	4.45 (1H, m)
		4.28 (1H, m)		4.29 (1H, m)		4.32 (1H, m)

Delta is ppm of chemical shifts which are reported in parts per million (δ), and coupling constants (*J*) are expressed in Hertz.

## Supplementary Materials

The following are available online. ^1^H-NMR, ^13^C-NMR, HSQC, HMBC, ^1^H-^1^H COSY, NOESY, HRESIMS, Infrared spectrum of compounds **1**–**5**.
