# Supplementary material for: Four Novel Dammarane-Type Triterpenoids from Pearl Knots of Panax ginseng Meyer cv. Silvatica
Source: Molecules. 2019 Mar 23;24(6):1159. doi: 10.3390/molecules24061159 (PMC6470847; doi:10.3390/molecules24061159)
Supplement: Supplementary file 1 [file molecules-24-01159-s001.pdf]

SUPPLEMENTARY MATERIAL

Figure S1-1. ^{1}H NMR spectrum of ginsengenin-S1 (**1**) in pyridine (600 MHz).
Figure S1-2. ^{13}C NMR spectrum of ginsengenin-S1 (**1**) in pyridine (150 MHz).
Figure S1-3. Heteronuclear single quantum correlation (HSQC) spectrum of ginsengenin-S1 (**1**) in pyridine.
Figure S1-4. Heteronuclear multiple bond correlation (HMBC) spectrum of ginsengenin-S1 (**1**) in pyridine.
Figure S1-5. ^{1}H-^{1}H correlation spectroscopy (^{1}H-^{1}H COSY) spectrum of ginsengenin-S1 (**1**) in pyridine.
Figure S1-6. Nuclear Overhauser effect spectroscopy (NOESY) spectrum of ginsengenin-S1 (**1**) in pyridine.
Figure S1-7. High resolution electrospray ionization mass spectroscopy (HRESIMS) spectrum of ginsengenin-S1 (**1**).
Figure S1-8. Infrared (IR) spectrum of ginsengenin-S1 (**1**).
Figure S2-1. ^{1}H NMR spectrum of ginsengenin-S2 (**2**) in pyridine (600 MHz).
Figure S2-2. ^{13}C NMR spectrum of ginsengenin-S2 (**2**) in pyridine (150 MHz).
Figure S2-3. Heteronuclear single quantum correlation (HSQC) spectrum of ginsengenin-S2 (**2**) in pyridine.
Figure S2-4. Heteronuclear multiple bond correlation (HMBC) spectrum of ginsengenin-S2 (**2**) in pyridine.
Figure S2-5. ^{1}H-^{1}H correlation spectroscopy (^{1}H-^{1}H COSY) spectrum of ginsengenin-S2 (**2**)in pyridine.
Figure S2-6. Nuclear Overhauser effect spectroscopy (NOESY) spectrum of ginsengenin-S2 (**2**) in pyridine.
Figure S2-7. High resolution electrospray ionization mass spectroscopy (HRESIMS) spectrum of ginsengenin-S2 (**2**).
Figure S2-8. Infrared (IR) spectrum of ginsengenin-S2 (**2**).
Figure S3-1. ^{1}H NMR spectrum of ginsenoside-S3 (**3**) in pyridine (600 MHz).
Figure S3-2. ^{13}C NMR spectrum of ginsenoside-S3 (**3**) in pyridine (150 MHz).
Figure S3-3. Heteronuclear single quantum correlation (HSQC) spectrum of ginsenoside-S3 (**3**) in pyridine.
Figure S3-4. Heteronuclear multiple bond correlation (HMBC) spectrum of ginsenoside-S3 (**3**) in pyridine.
Figure S3-5. High resolution electrospray ionization mass spectroscopy (HRESIMS) spectrum of ginsenoside-S3 (**3**).
Figure S3-6. Infrared (IR) spectrum of ginsenoside-S3 (**3**).
Figure S4-1. ^{1}H NMR spectrum of ginsenoside-S4 (**4**) in pyridine (600 MHz).
Figure S4-2. ^{13}C NMR spectrum of ginsenoside-S4 (**4**) in pyridine (150 MHz).
Figure S4-3. Heteronuclear single quantum correlation (HSQC) spectrum of ginsenoside-S4 (**4**) in pyridine.
Figure S4-4. Heteronuclear multiple bond correlation (HMBC) spectrum of ginsenoside-S4 (**4**) in pyridine.

Figure S4-5. High resolution electrospray ionization mass spectroscopy (HRESIMS) spectrum of ginsenoside-S4 (**4**).
Figure S4-6. Infrared (IR) spectrum of ginsenoside-S4 (**4**).
Figure S5-1. ^{1}H NMR spectrum of ginsenoside-S5 (**5**) in pyridine (600 MHz).
Figure S5-2. ^{13}C NMR spectrum of ginsenoside-S5 (**5**) in pyridine (150 MHz).
Figure S5-3. Heteronuclear single quantum correlation (HSQC) spectrum of ginsenoside-S5 (**5**) in pyridine.
Figure S5-4. Heteronuclear multiple bond correlation (HMBC) spectrum of ginsenoside-S5 (**5**) in pyridine.
Figure S5-5. High resolution electrospray ionization mass spectroscopy (HRESIMS) spectrum of ginsenoside-S5 (**5**).
Figure S5-6. Infrared (IR) spectrum of ginsenoside-S5 (**5**).

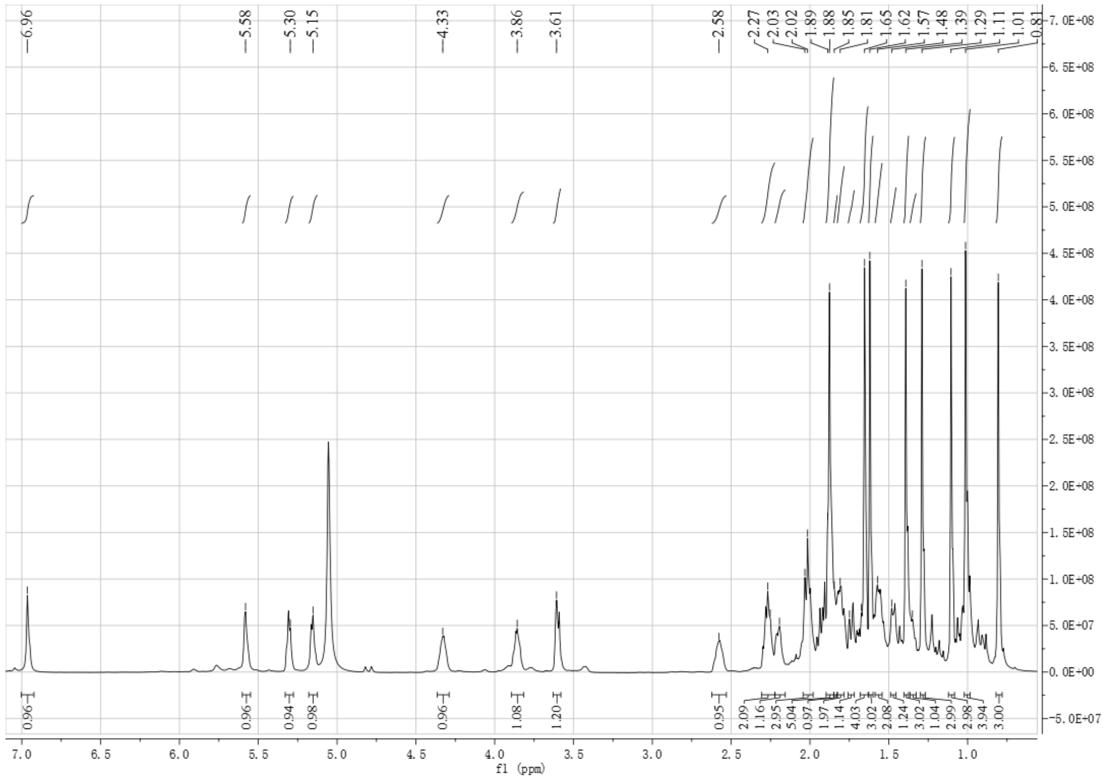

Figure S1-1. ^{1}H NMR spectrum of ginsengenin-S1 (**1**) in pyridine (600MHz).

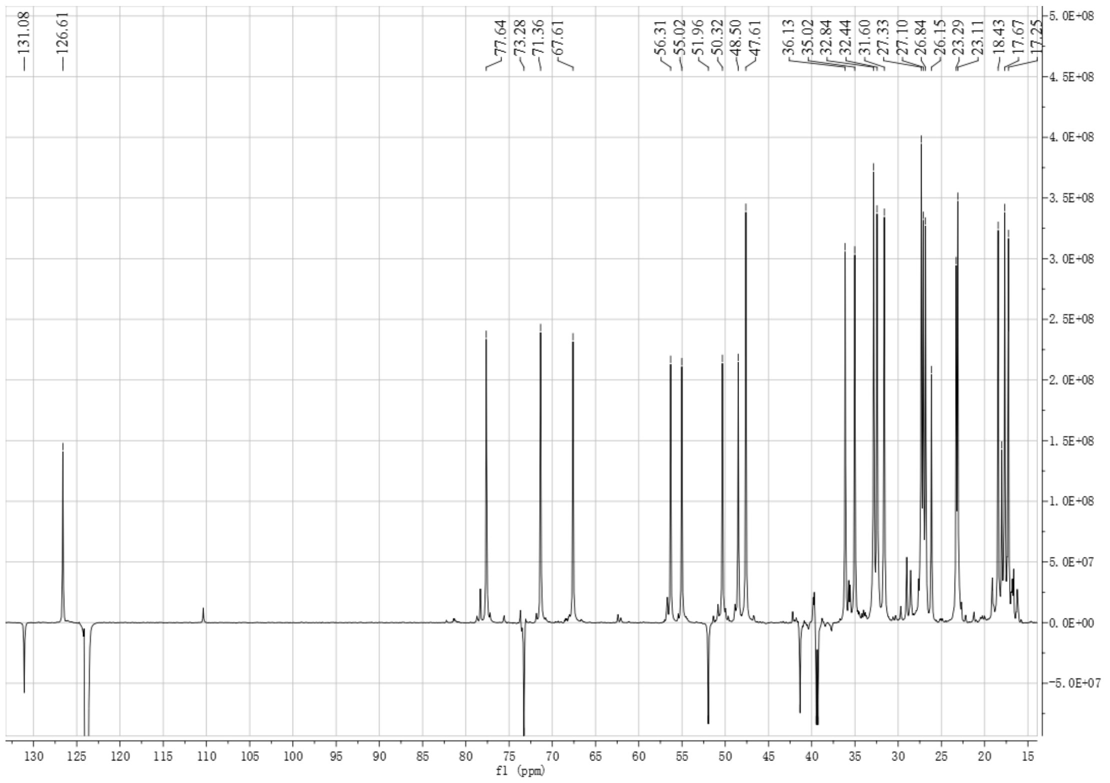

Figure S1-2. ^{13}C NMR spectrum of ginsengenin-S1 (**1**) in pyridine (150MHz).

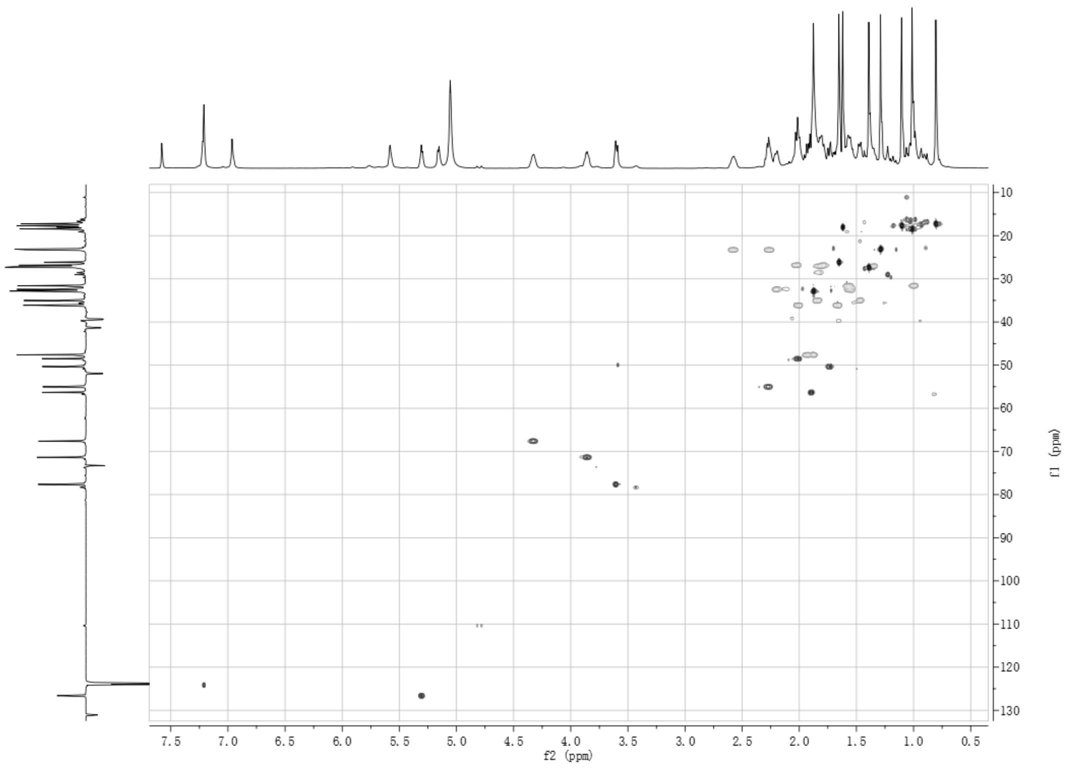

Figure S1-3. Heteronuclear single quantum correlation (HSQC) spectrum of ginsengenin-S1 (**1**) in pyridine.

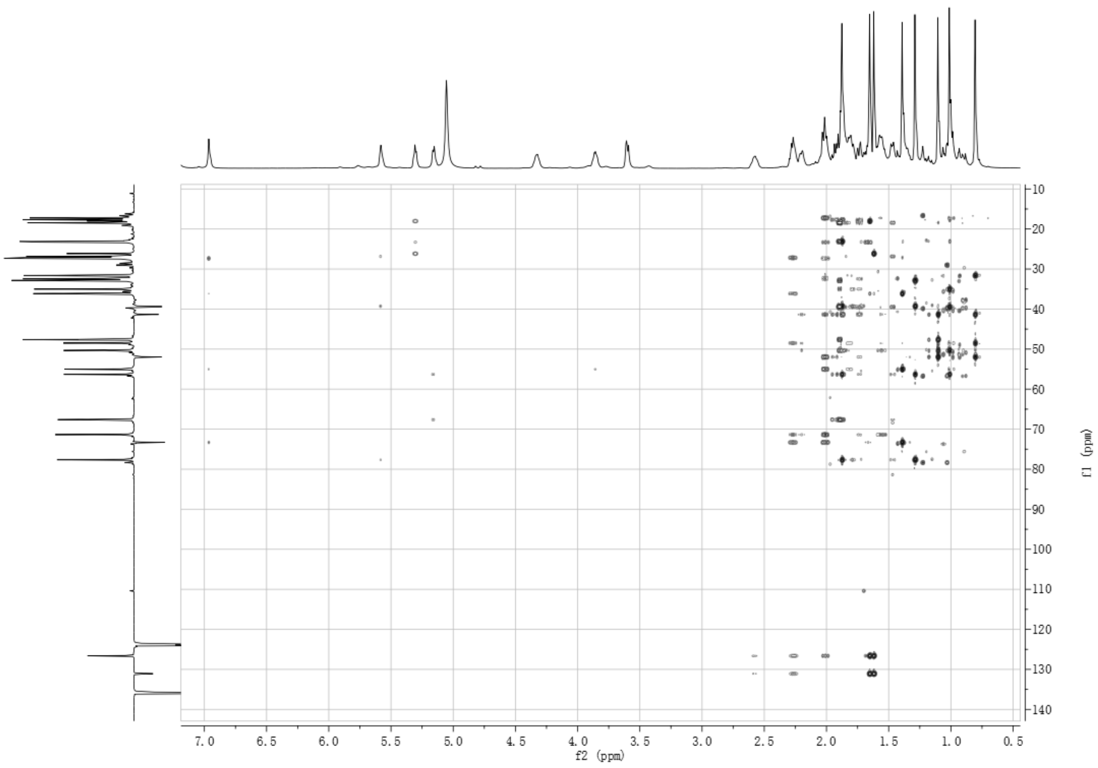

Figure S1-4. Heteronuclear multiple bond correlation (HMBC) spectrum of ginsengenin-S1 (**1**) in pyridine.

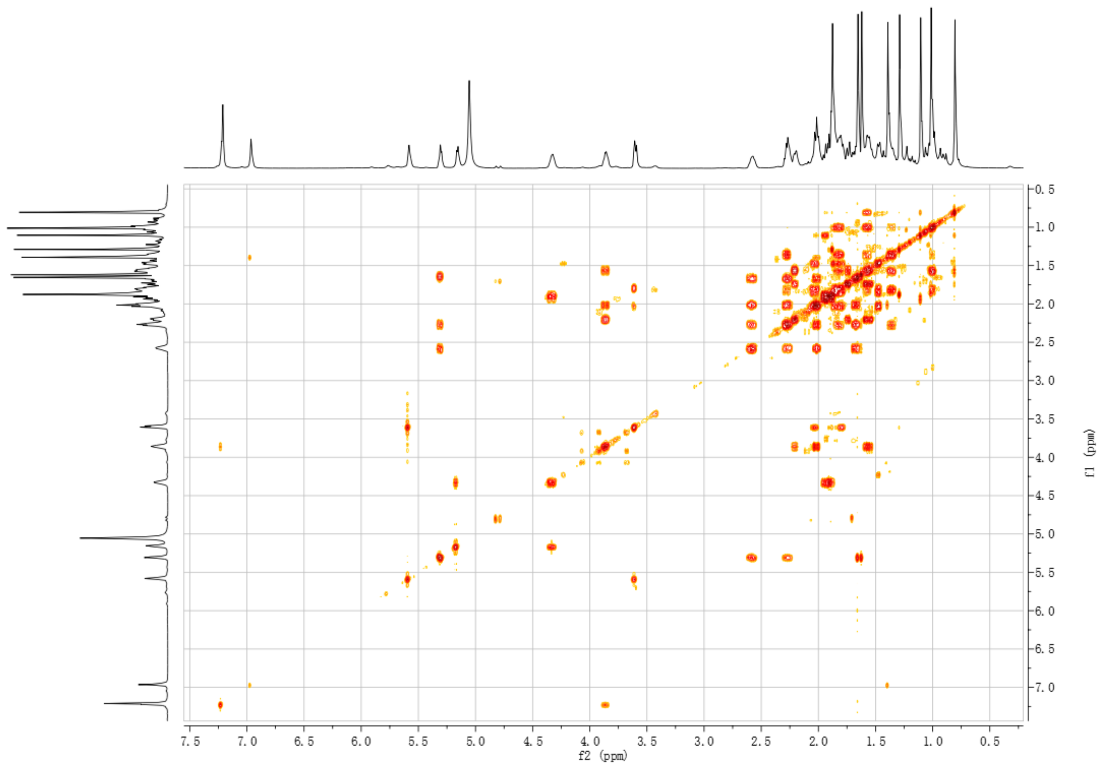

Figure S1-5. ^{1}H-^{1}H correlation spectroscopy (^{1}H-^{1}H COSY) spectrum of ginsengenin-S1 (**1**) in pyridine.

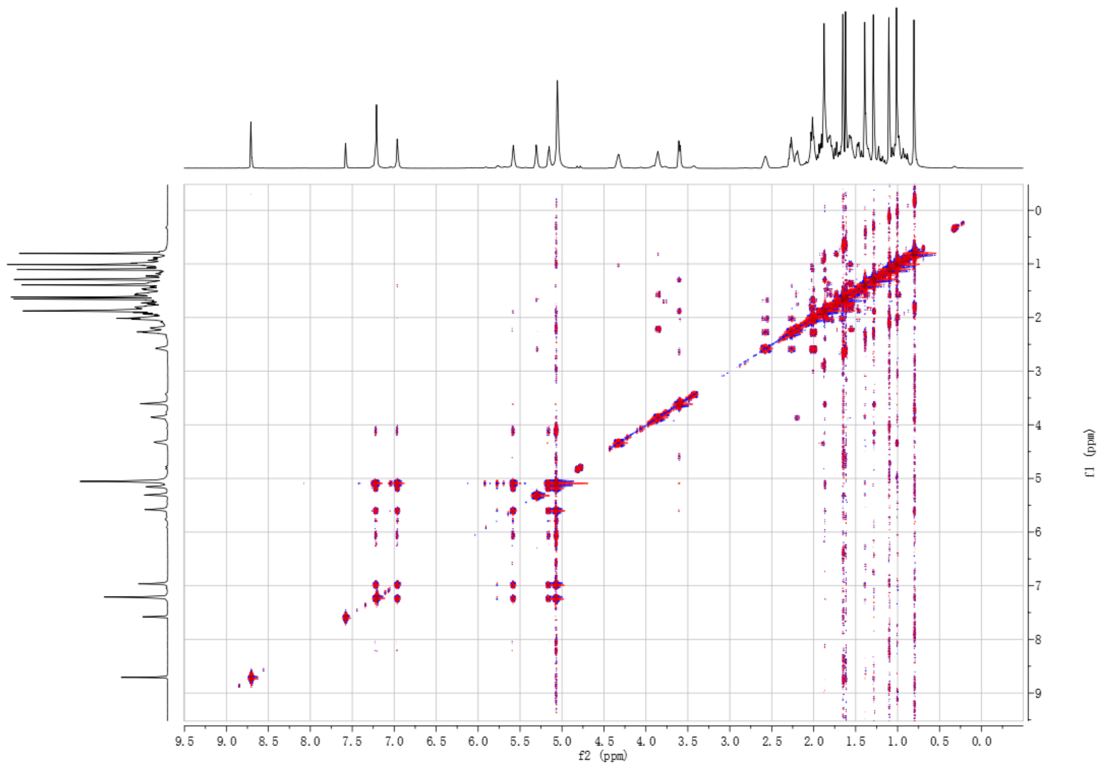

Figure S1-6. Nuclear Overhauser effect spectroscopy (NOESY) spectrum of ginsengenin-S1 (**1**) in pyridine.

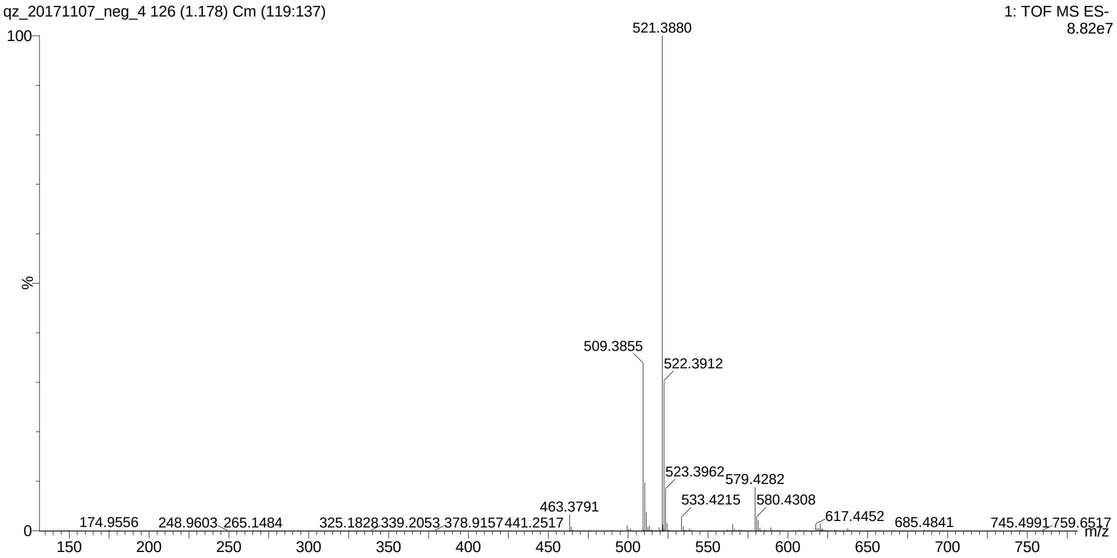

Figure S1-7. High resolution electrospray ionization mass spectroscopy (HRESIMS) spectrum of ginsengenin-S1 (**1**).

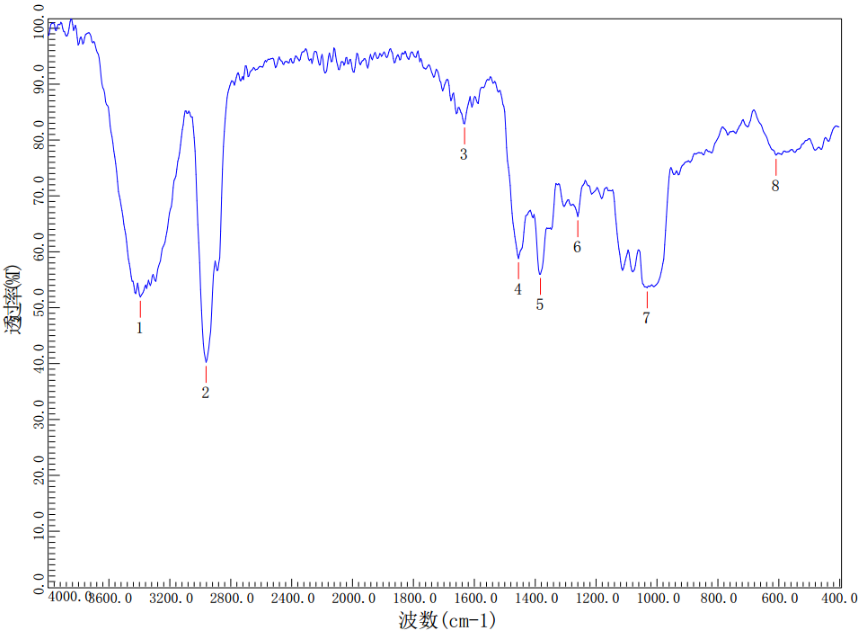

序号	波数(cm-1)	峰值
1	3394.0	51.9
2	2962.0	40.2
3	1632.0	82.9
4	1455.0	58.8
5	1383.0	56.0
6	1260.0	66.3
7	1032.0	53.6
8	609.0	77.3

Figure S1-8. Infrared (IR) spectrum of ginsengenin-S1 (**1**).

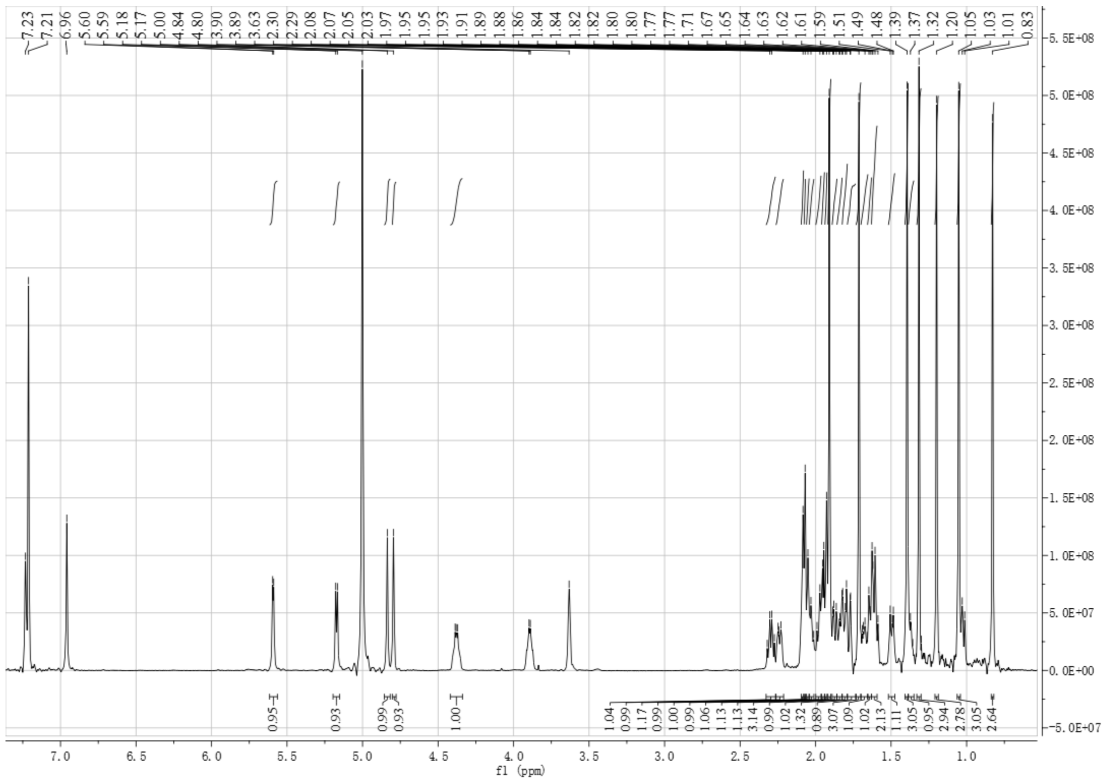

Figure S2-1. ^{1}H NMR spectrum of ginsengenin-S2 (**2**) in pyridine (600MHz).

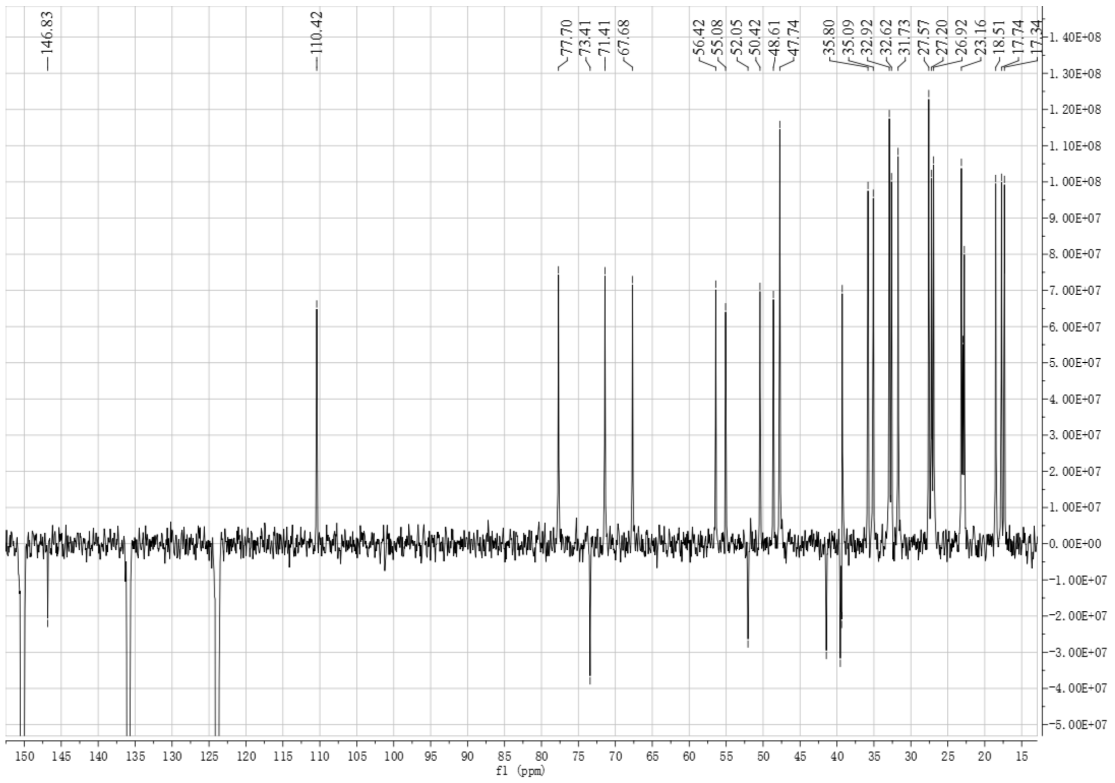

Figure S2-2. ^{13}C NMR spectrum of ginsengenin-S2 (**2**) in pyridine (150MHz).

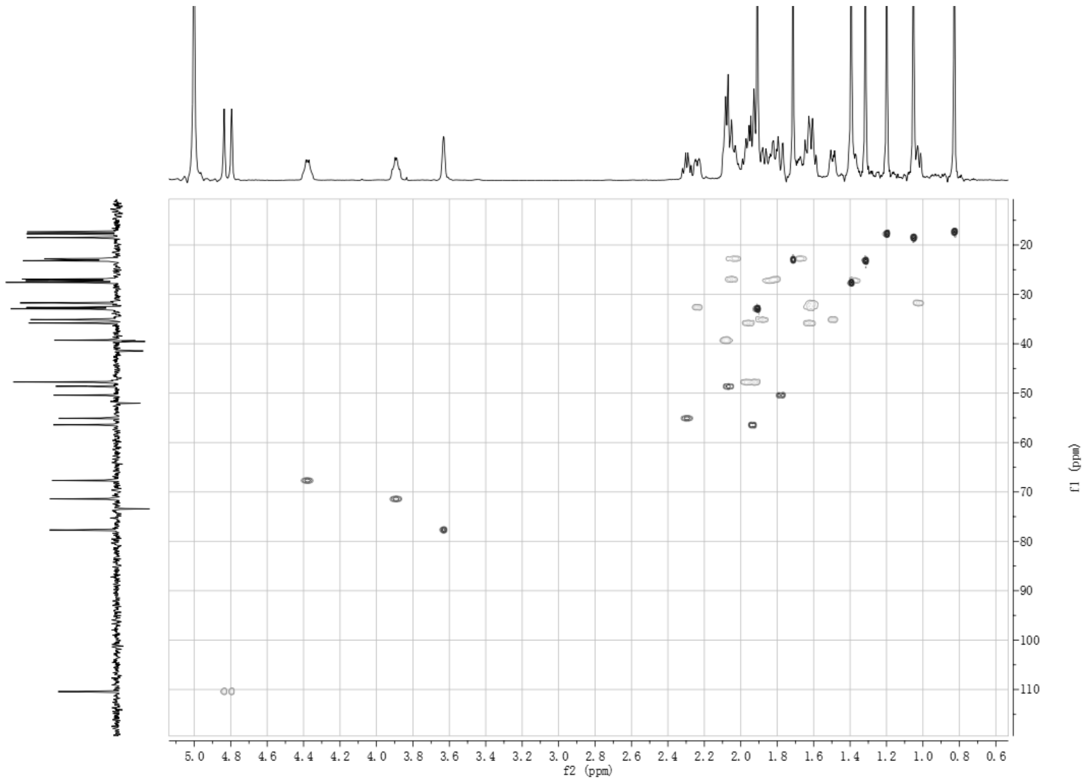

Figure S2-3. Heteronuclear single quantum correlation (HSQC) spectrum of ginsengenin-S2 (**2**) in pyridine.

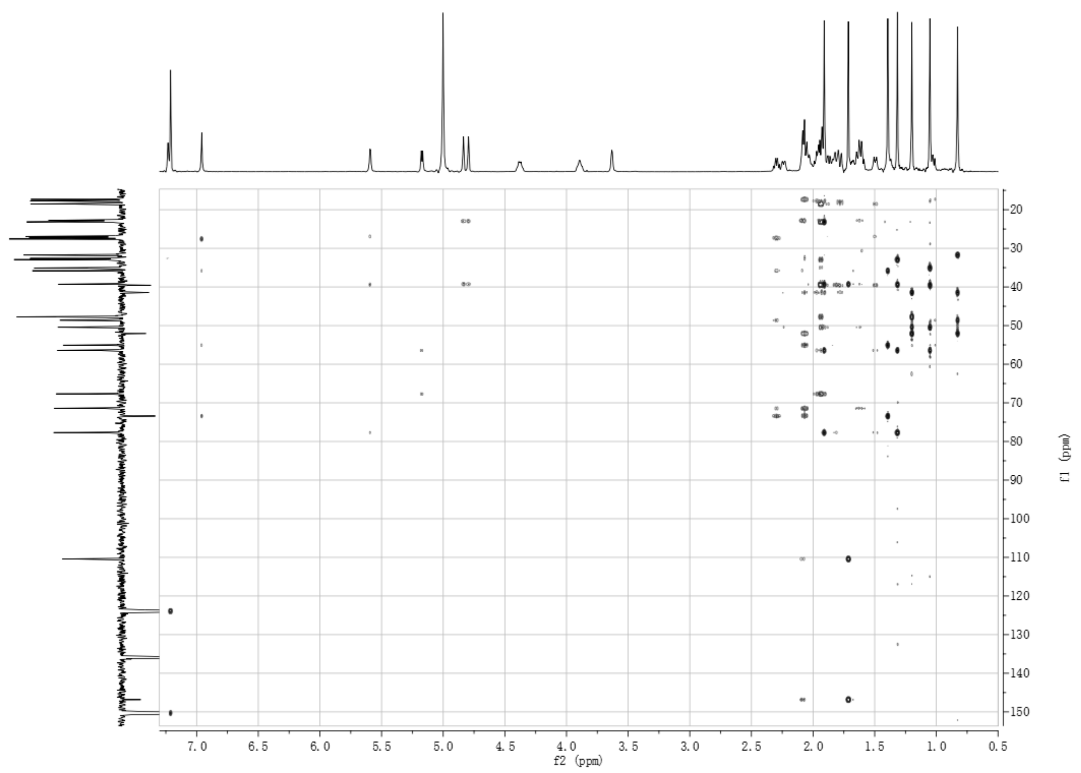

Figure S2-4. Heteronuclear multiple bond correlation (HMBC) spectrum of ginsengenin-S2 (**2**) in pyridine.

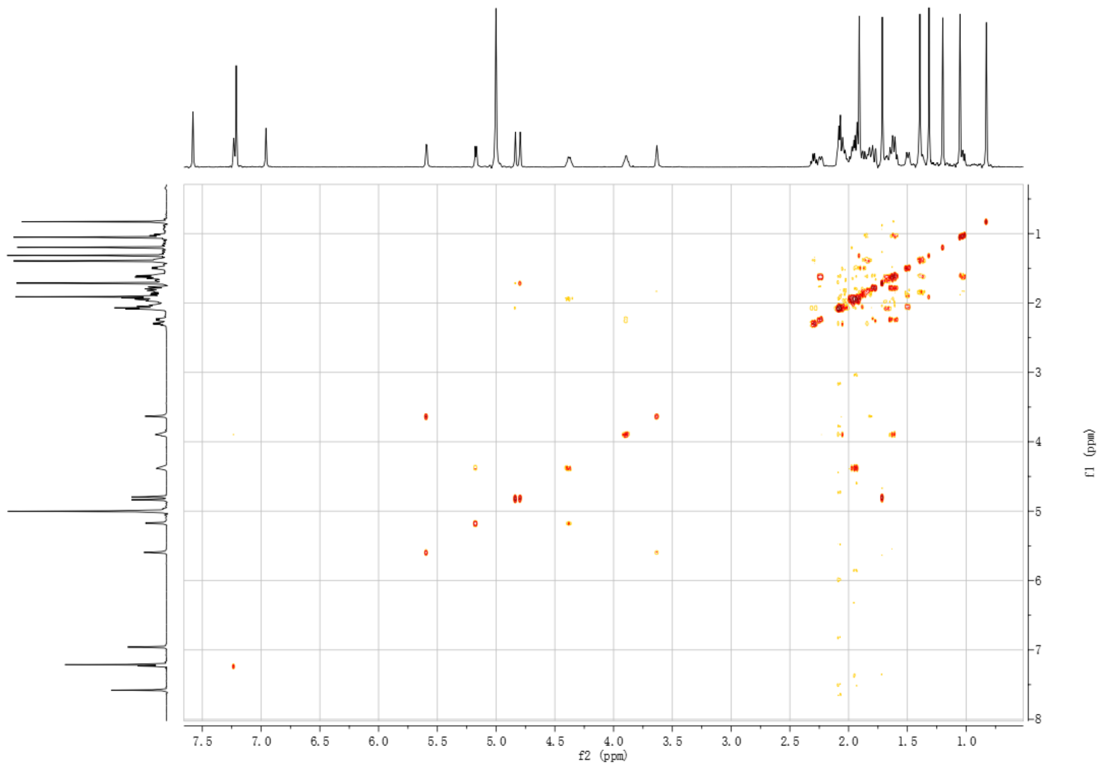

Figure S2-5. ^{1}H-^{1}H correlation spectroscopy (^{1}H-^{1}H COSY) spectrum of ginsengenin-S2 (**2**)in pyridine.

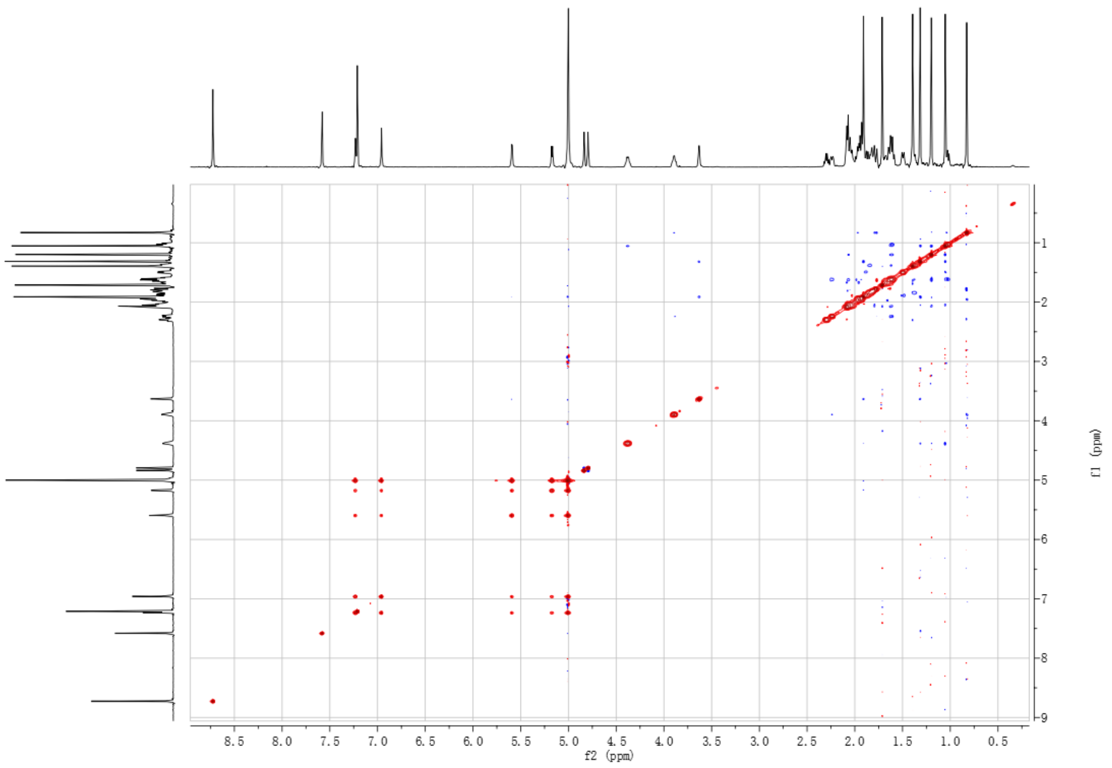

Figure S2-6. Nuclear Overhauser effect spectroscopy (NOESY) spectrum of ginsengenin-S2 (**2**) in pyridine.

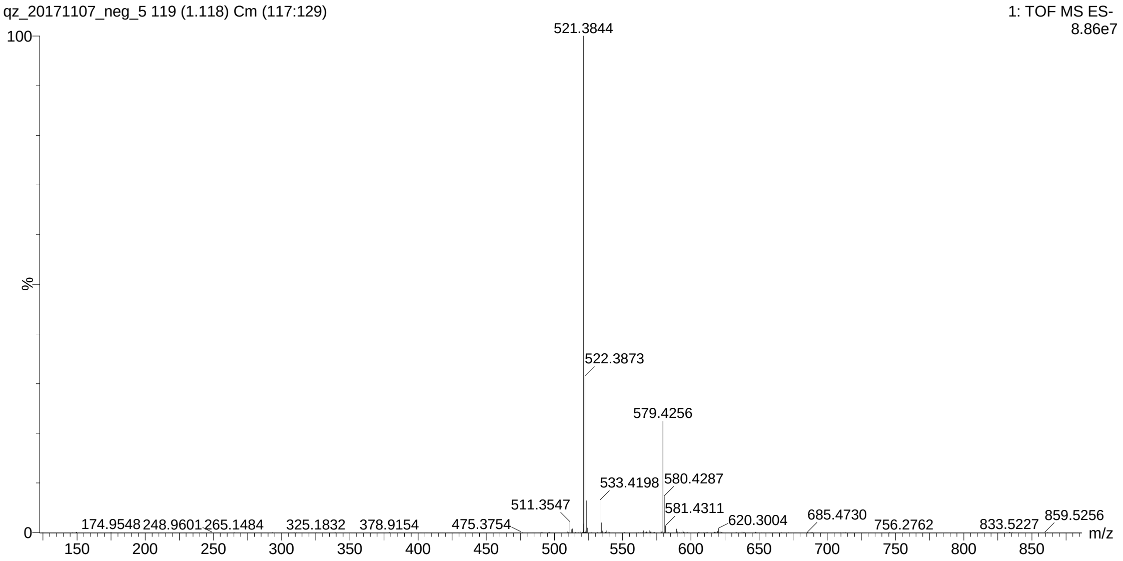

Figure S2-7. High resolution electrospray ionization mass spectroscopy (HRESIMS) spectrum of ginsengenin-S2 (**2**).

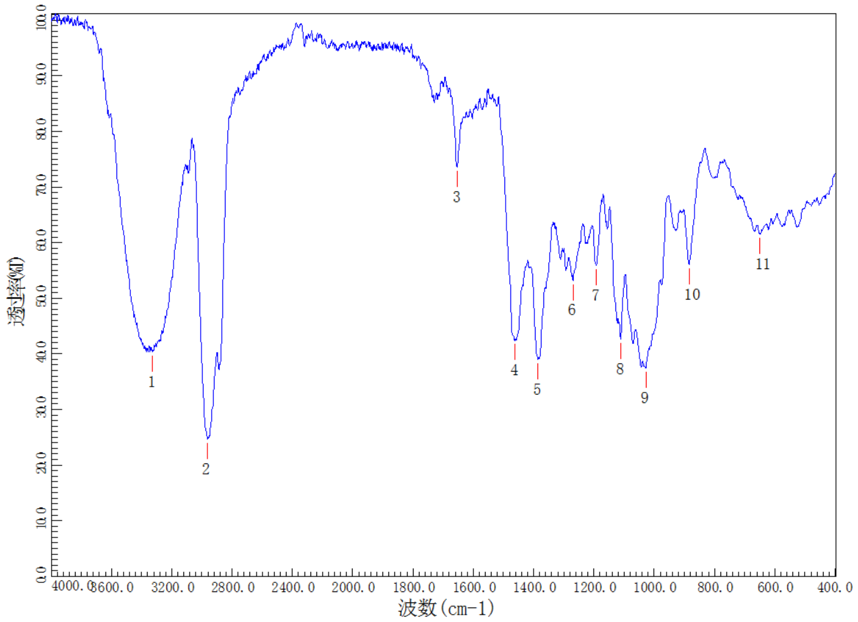

序号	波数(cm-1)	峰值
1	3328.0	40.3
2	2964.0	24.6
3	1653.0	73.5
4	1461.0	42.4
5	1386.0	38.9
6	1269.0	53.2
7	1191.0	55.9
8	1111.0	42.6
9	1028.0	37.5
10	884.0	56.0
11	649.0	61.5

Figure S2-8. Infrared (IR) spectrum of ginsengenin-S2 (**2**).

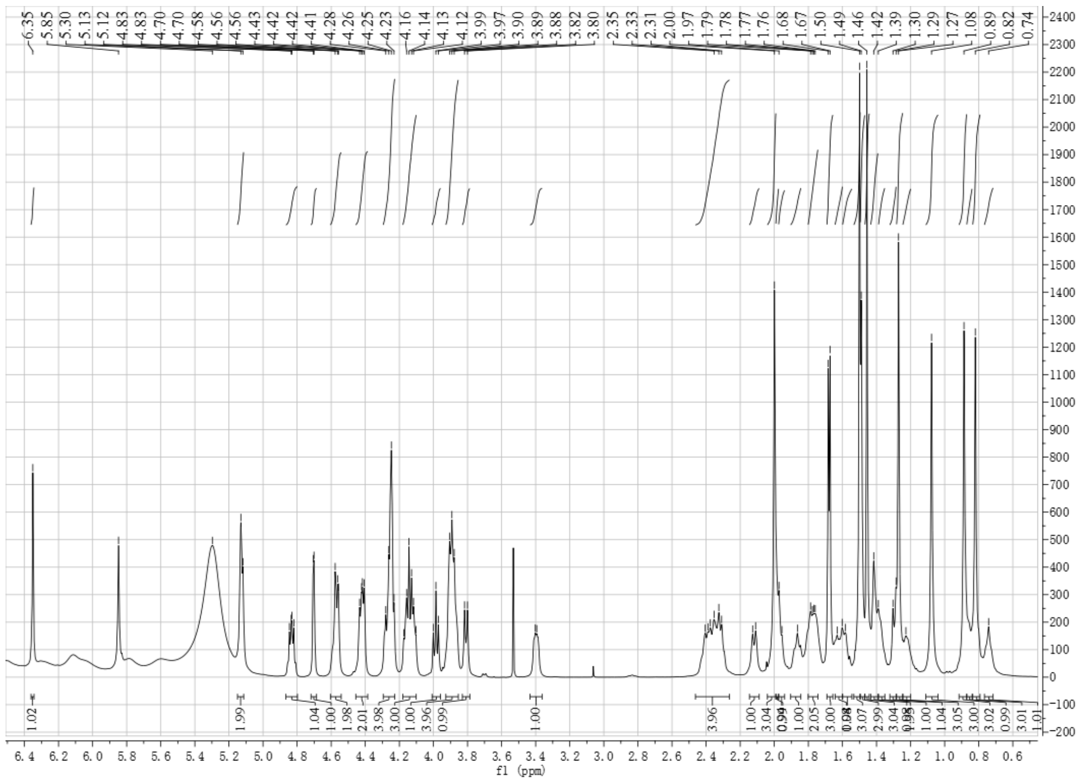

Figure S3-1. ^{1}H NMR spectrum of ginsenoside-S3 (**3**) in pyridine (600MHz).

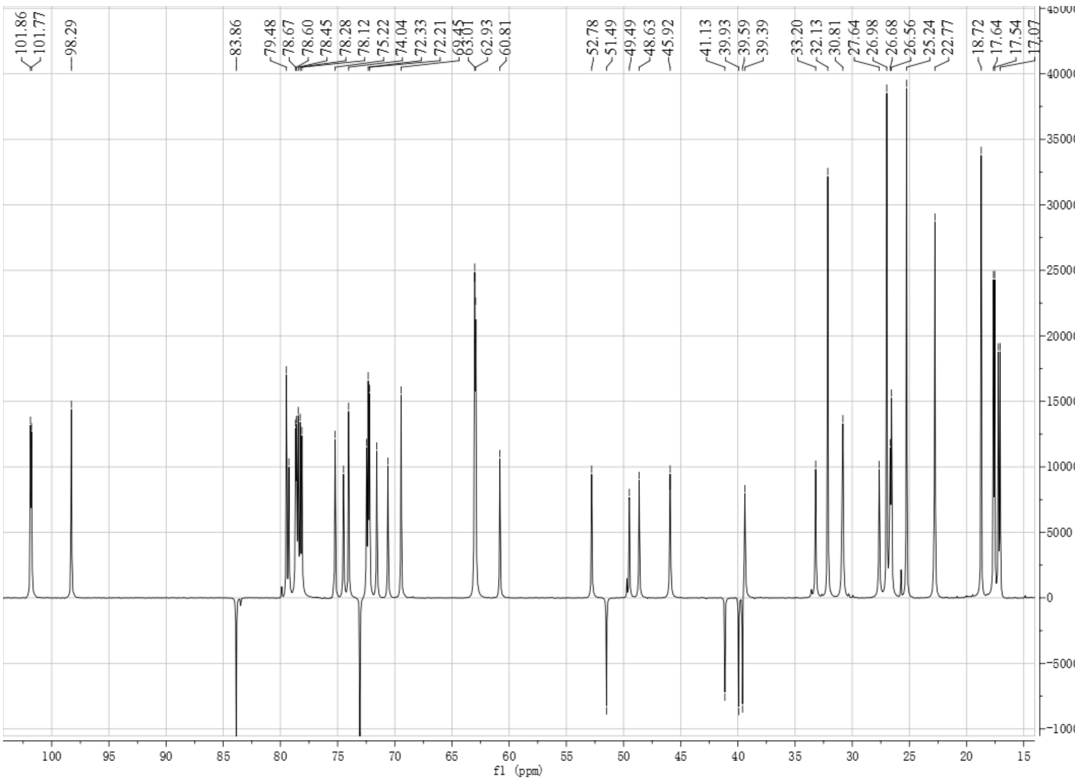

Figure S3-2. ^{13}C NMR spectrum of ginsenoside-S3 (**3**) in pyridine (150MHz).

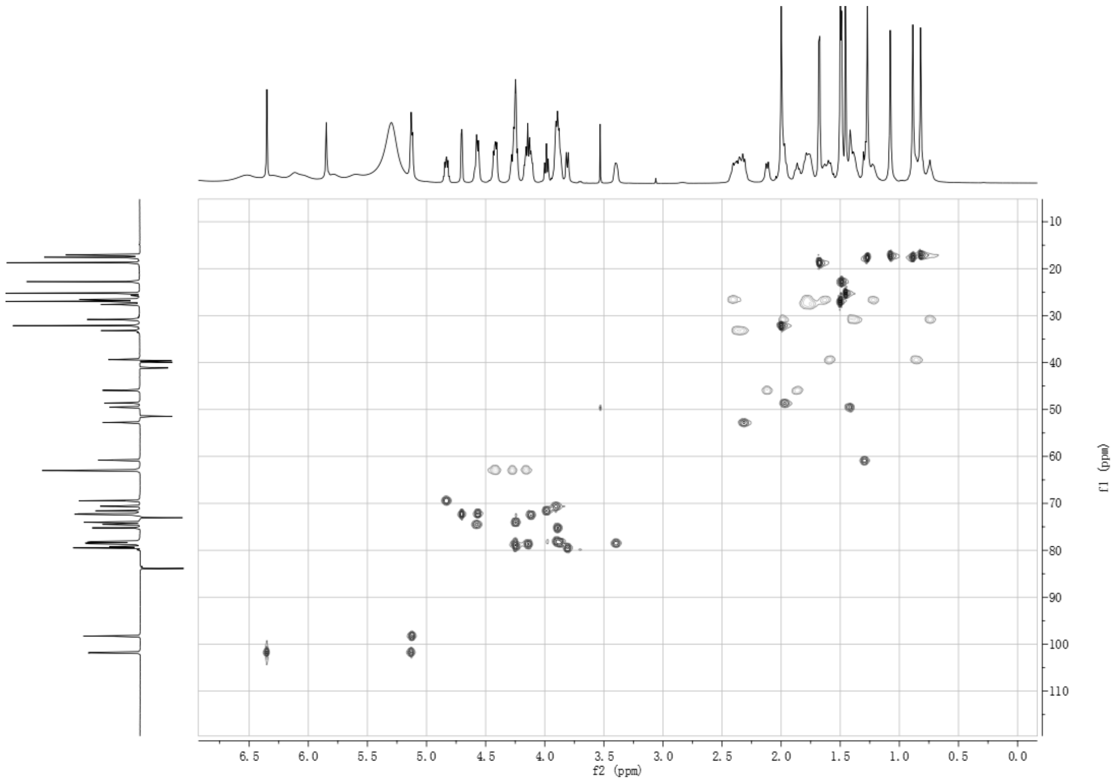

Figure S3-3. Heteronuclear single quantum correlation (HSQC) spectrum of ginsenoside-S3 (**3**) in pyridine.

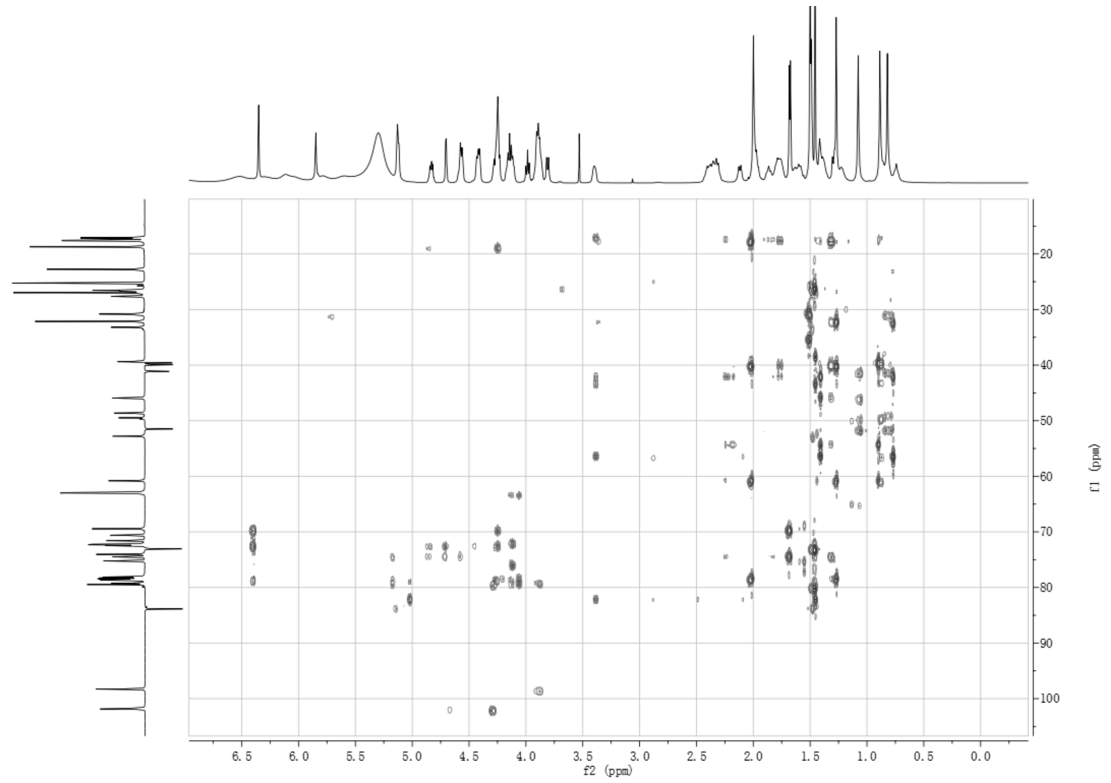

Figure S3-4. Heteronuclear multiple bond correlation (HMBC) spectrum of ginsenoside-S3 (**3**) in pyridine.

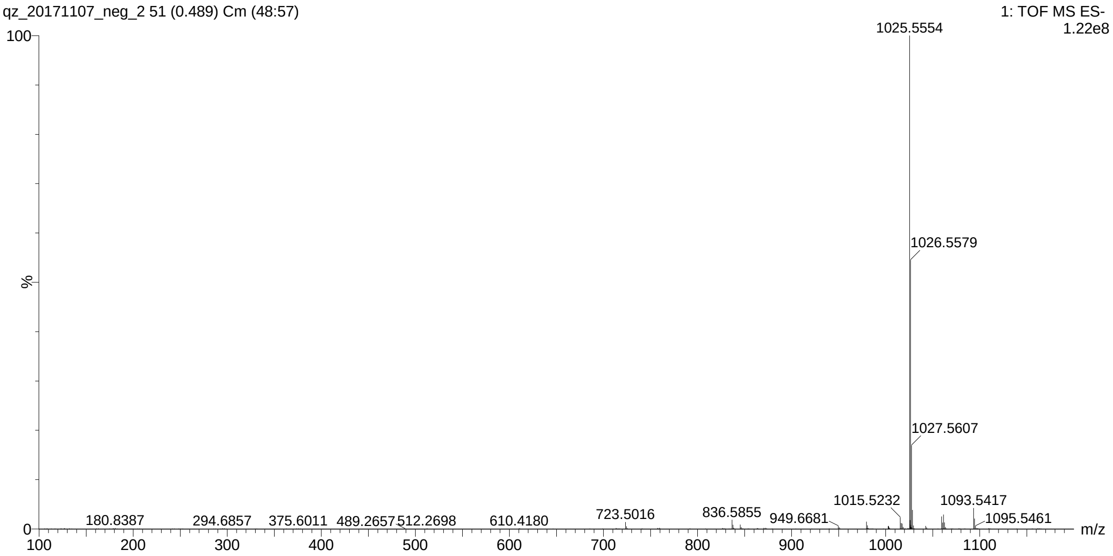

Figure S3-5. High resolution electrospray ionization mass spectroscopy (HRESIMS) spectrum of ginsenoside-S3 (**3**).

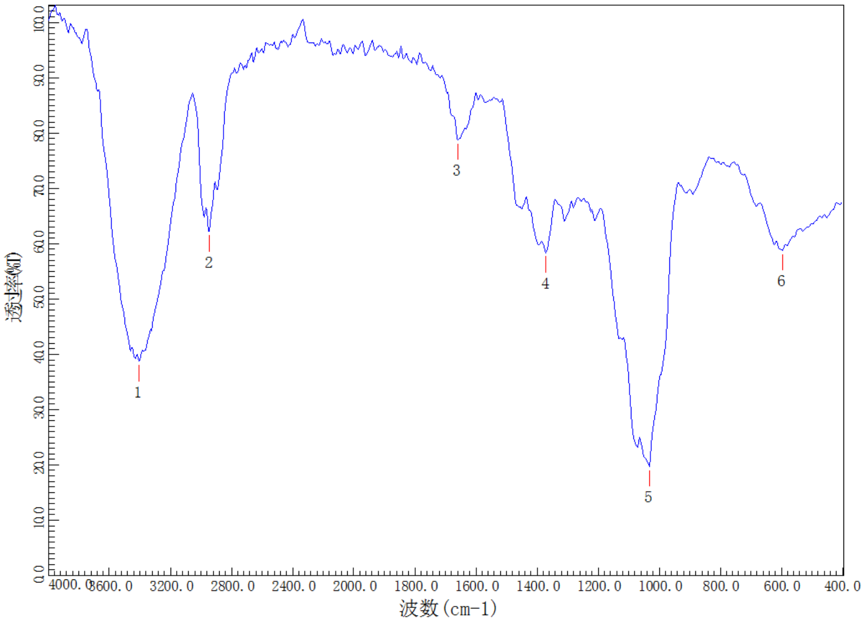

序号	波数(cm-1)	峰值
1	3406.0	38.7
2	2944.0	62.2
3	1659.0	78.8
4	1371.0	58.4
5	1032.0	19.7
6	597.0	58.8

Figure S3-6. Infrared (IR) spectrum of ginsenoside-S3 (**3**).

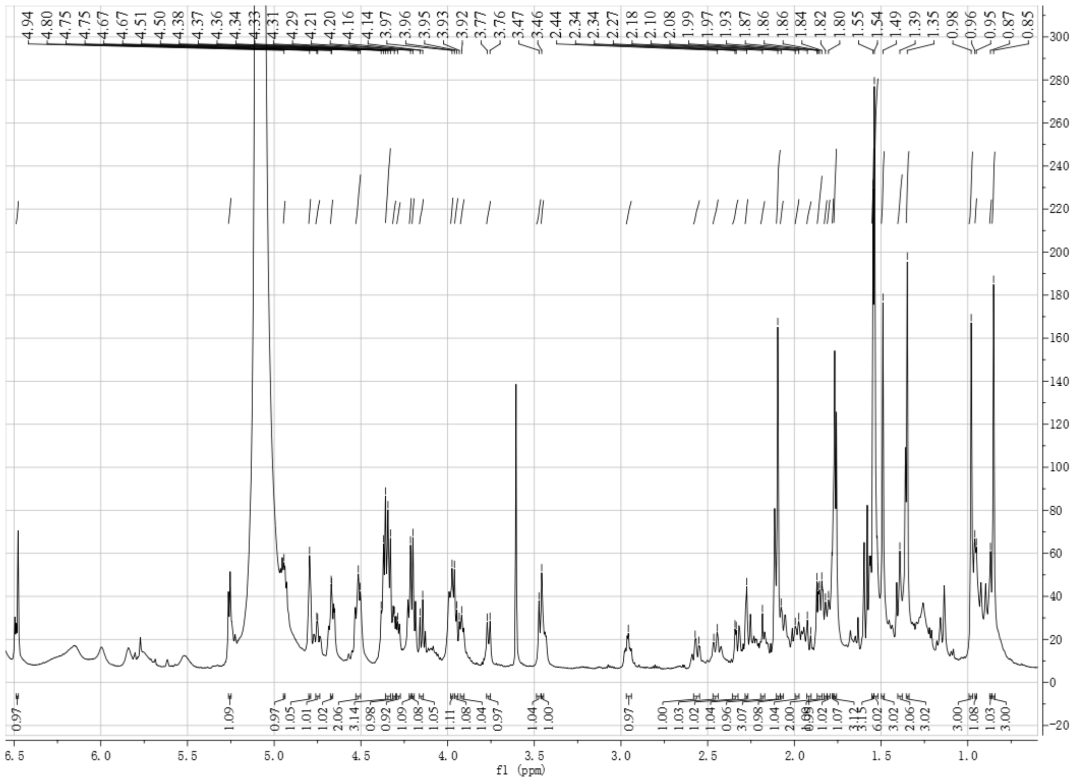

Figure S4-1. ^{1}H NMR spectrum of ginsenoside-S4 (**4**) in pyridine (600MHz).

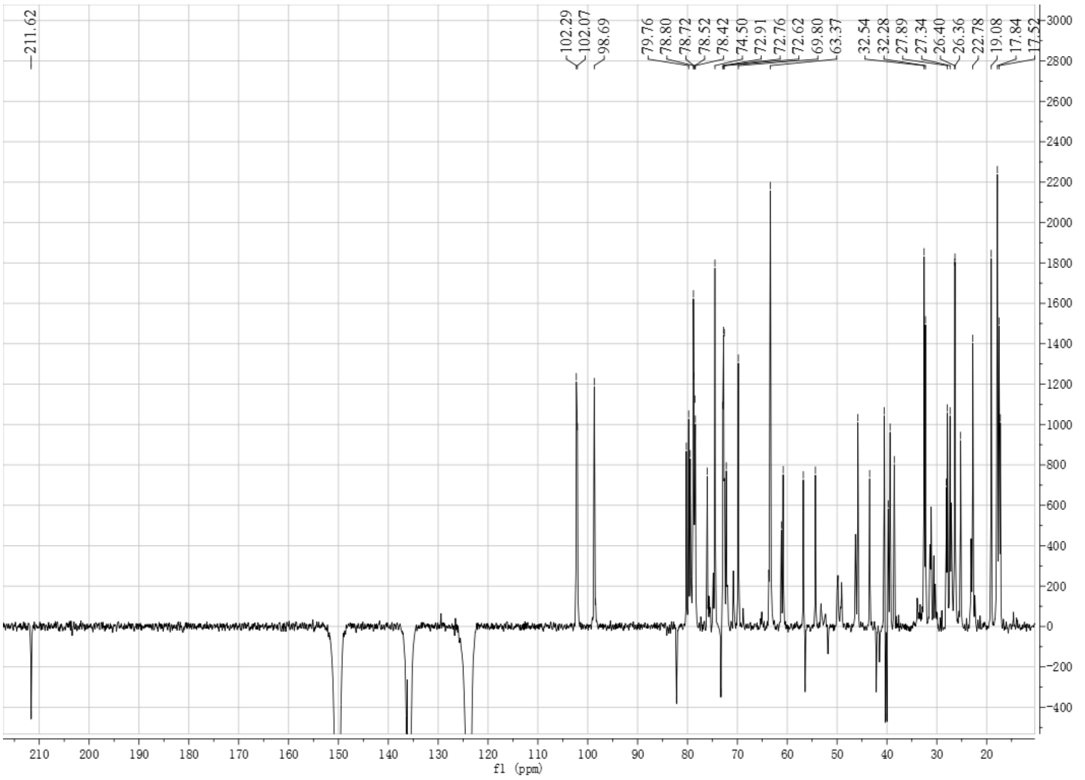

Figure S4-2. ^{13}C NMR spectrum of ginsenoside-S4 (**4**) in pyridine (150MHz).

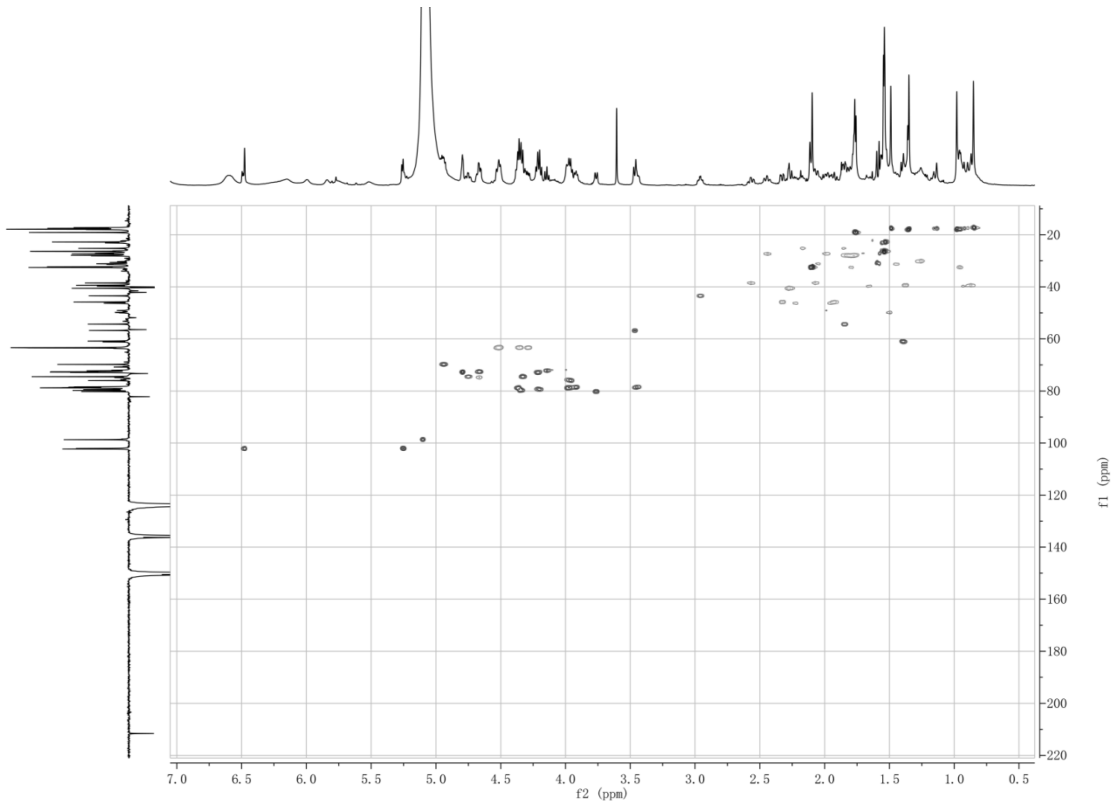

Figure S4-3. Heteronuclear single quantum correlation (HSQC) spectrum of ginsenoside-S4 (**4**) in pyridine.

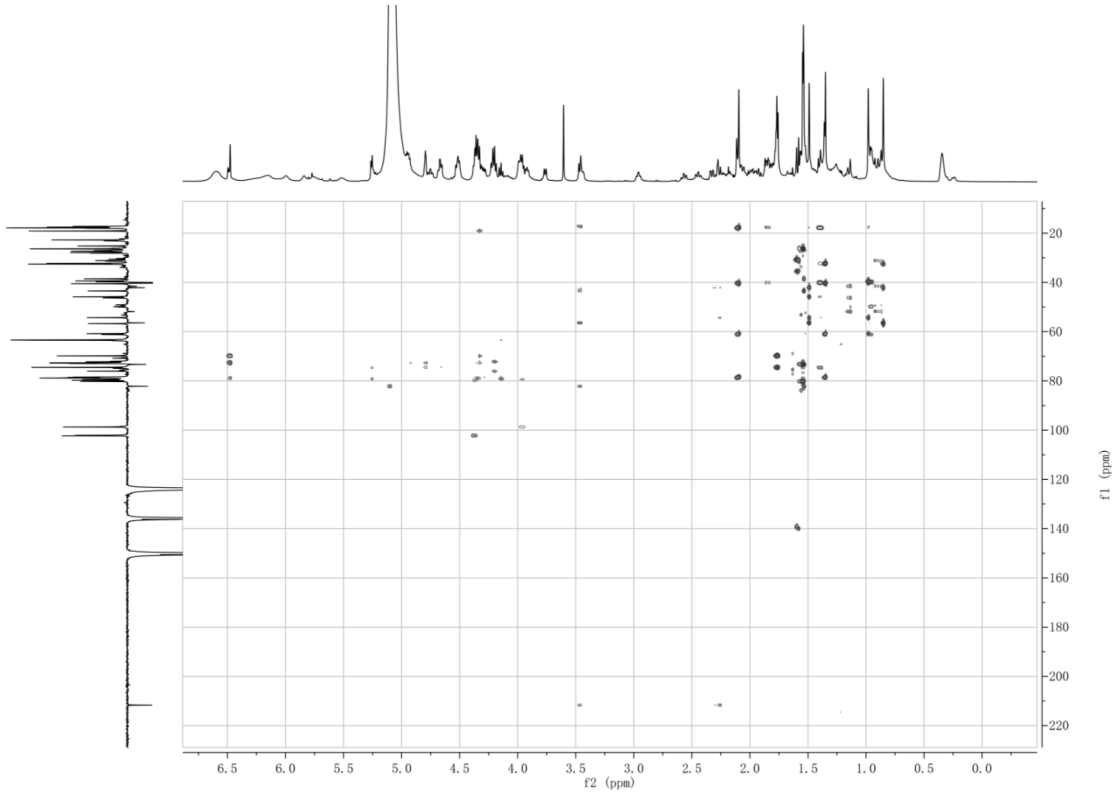

Figure S4-4. Heteronuclear multiple bond correlation (HMBC) spectrum of ginsenoside-S4 (**4**) in pyridine.

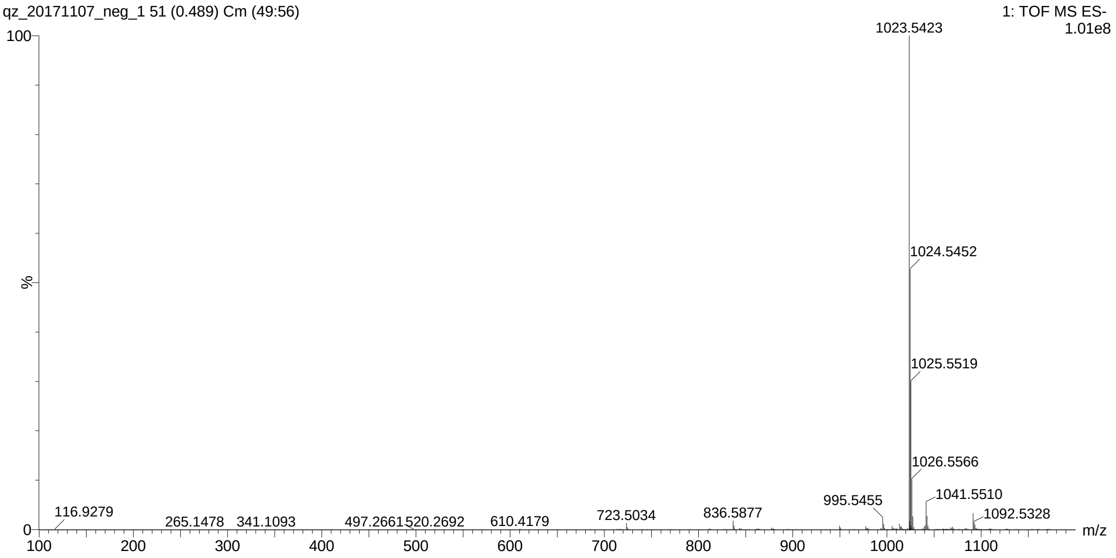

Figure S4-5. High resolution electrospray ionization mass spectroscopy (HRESIMS) spectrum of ginsenoside-S4 (**4**).

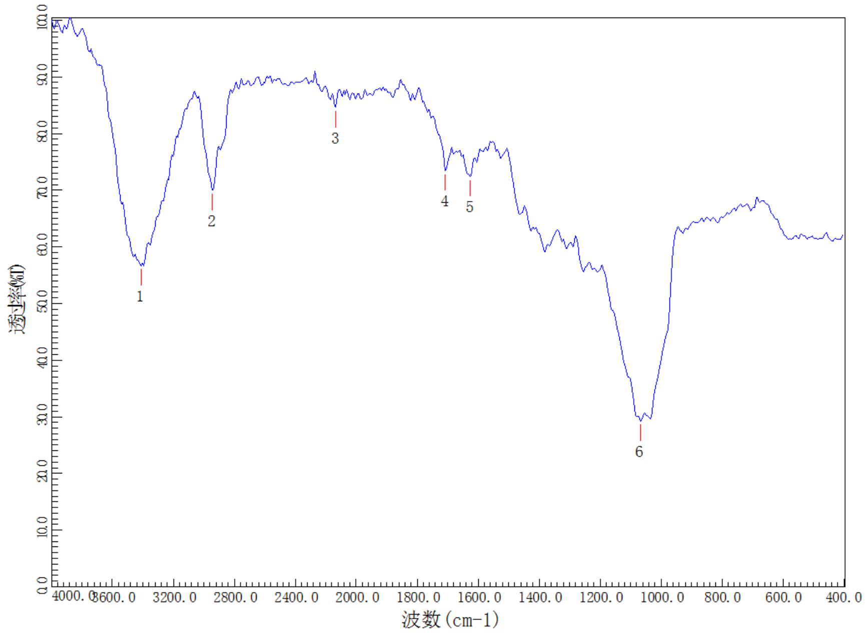

序号	波数(cm-1)	峰值
1	3412.0	56.7
2	2944.0	70.0
3	2134.0	84.6
4	1707.0	73.5
5	1626.0	72.5
6	1068.0	29.3

Figure S4-6. Infrared (IR) spectrum of ginsenoside-S4 (**4**).

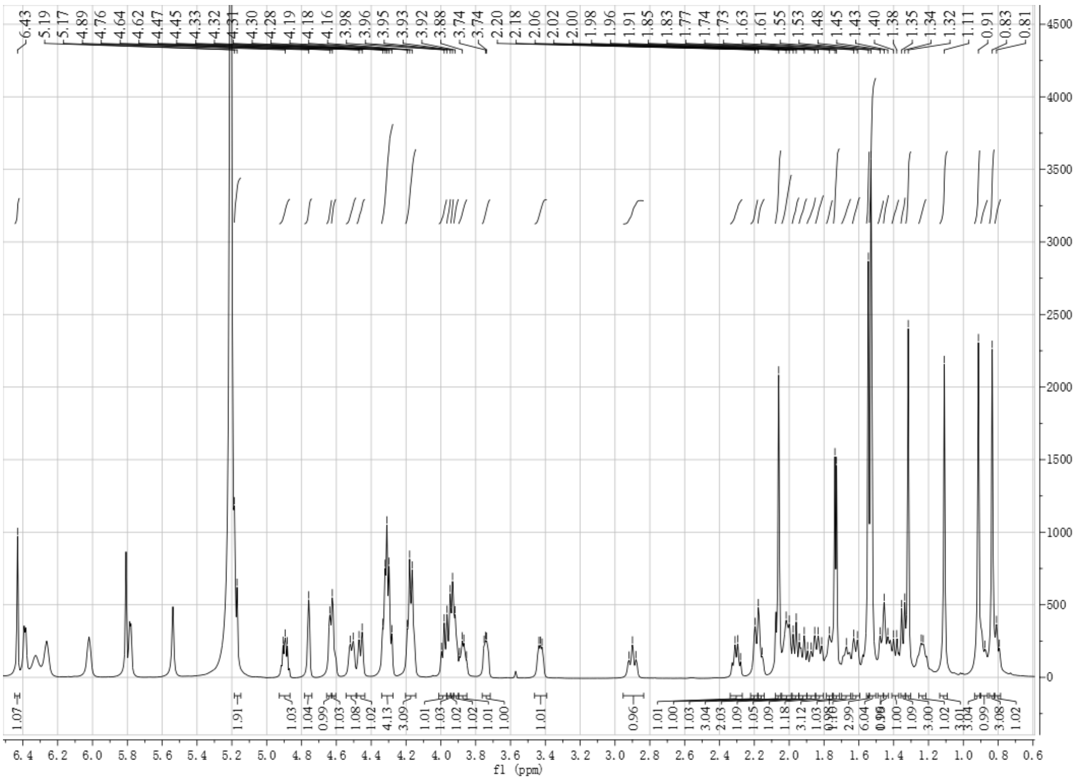

Figure S5-1. ^{1}H NMR spectrum of ginsenoside-S5 (**5**) in pyridine (600MHz).

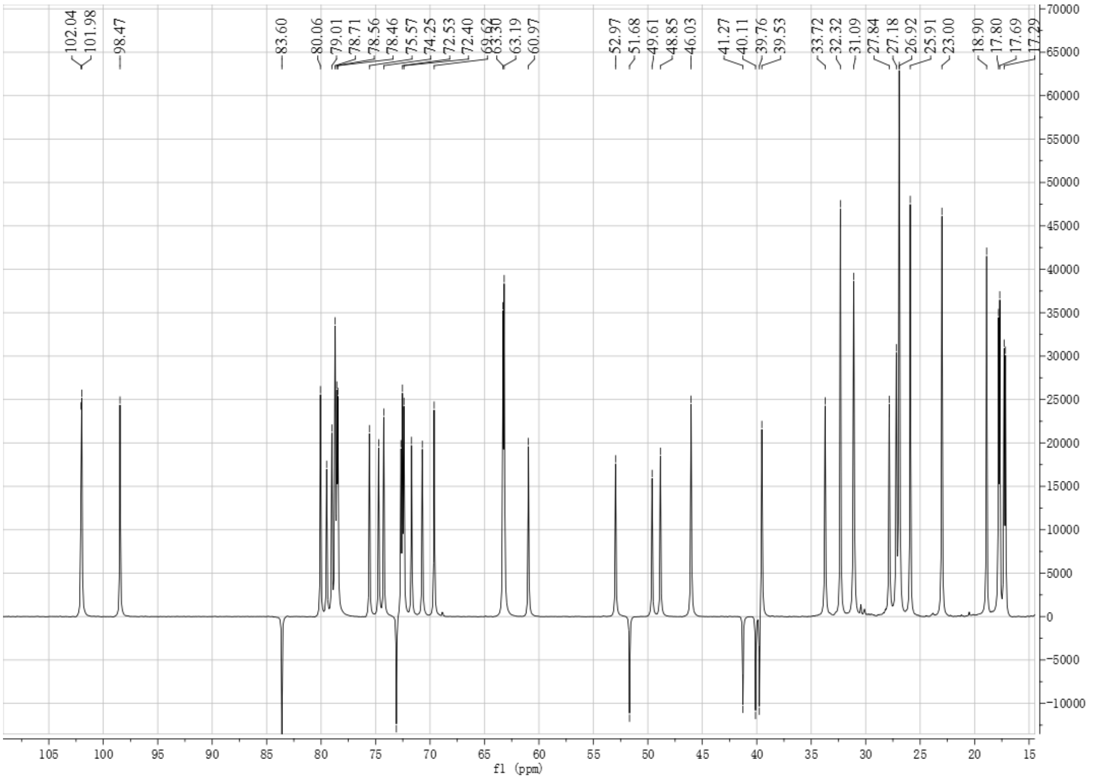

Figure S5-2. ^{13}C NMR spectrum of ginsenoside-S5 (**5**) in pyridine (150MHz).

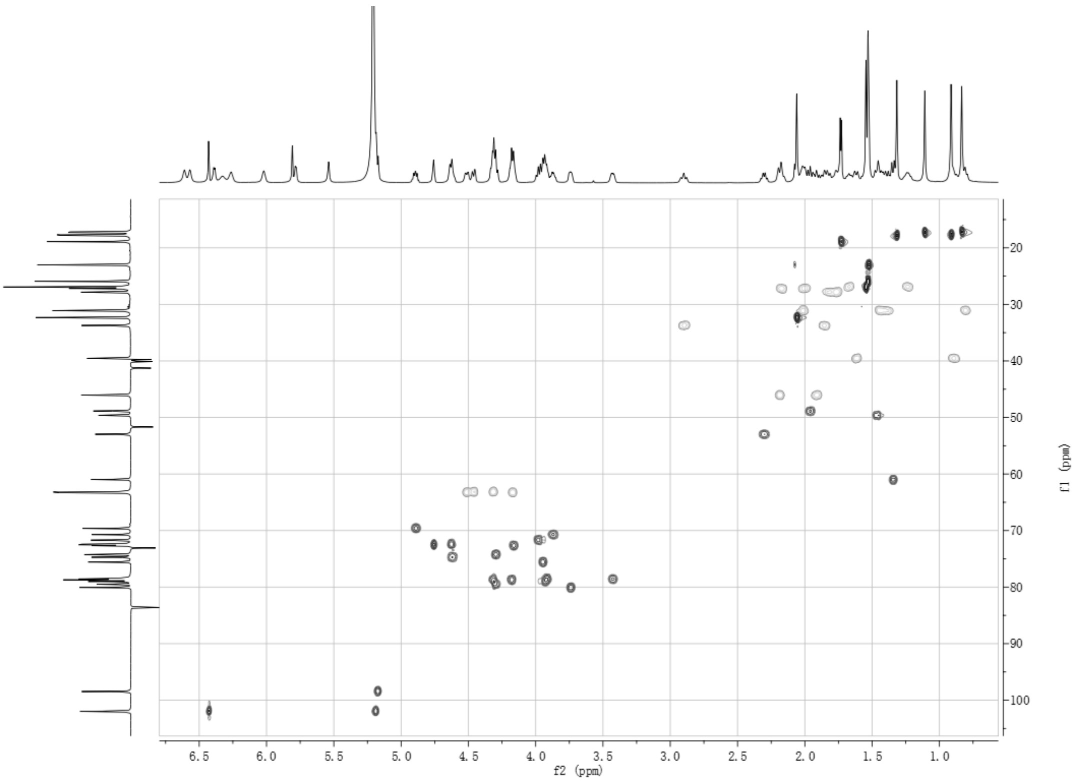

Figure S5-3. Heteronuclear single quantum correlation (HSQC) spectrum of ginsenoside-S5 (**5**) in pyridine.

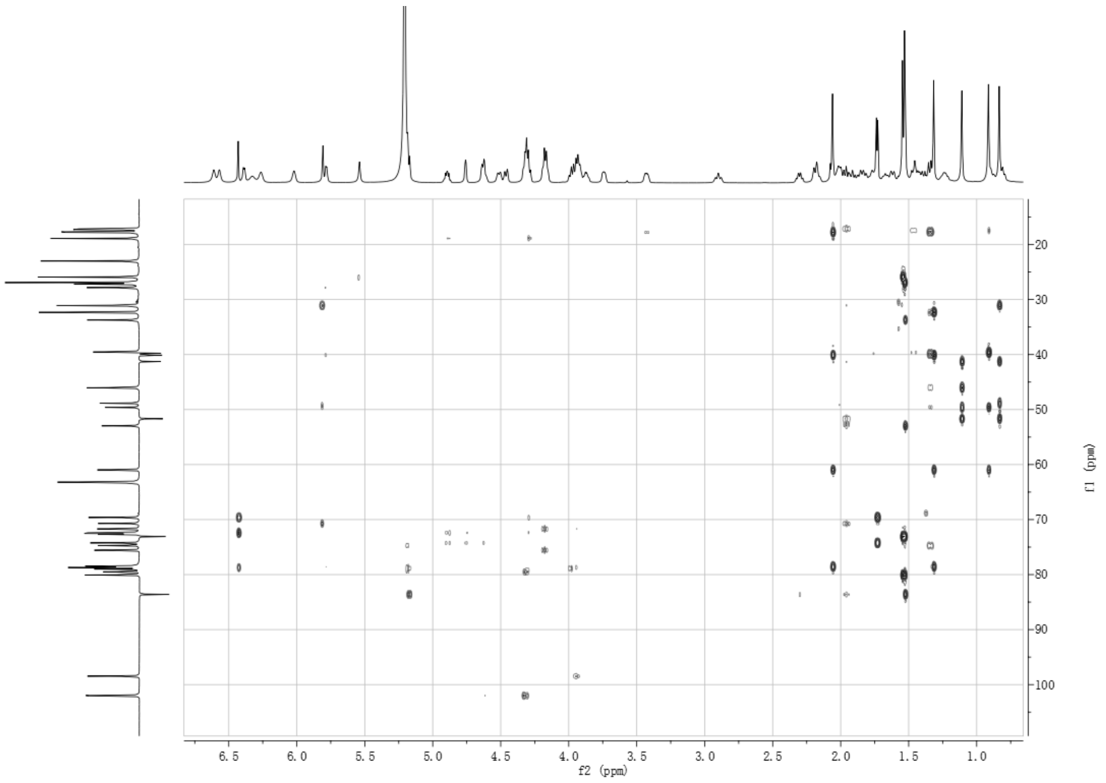

Figure S5-4. Heteronuclear multiple bond correlation (HMBC) spectrum of ginsenoside-S5 (**5**) in pyridine.

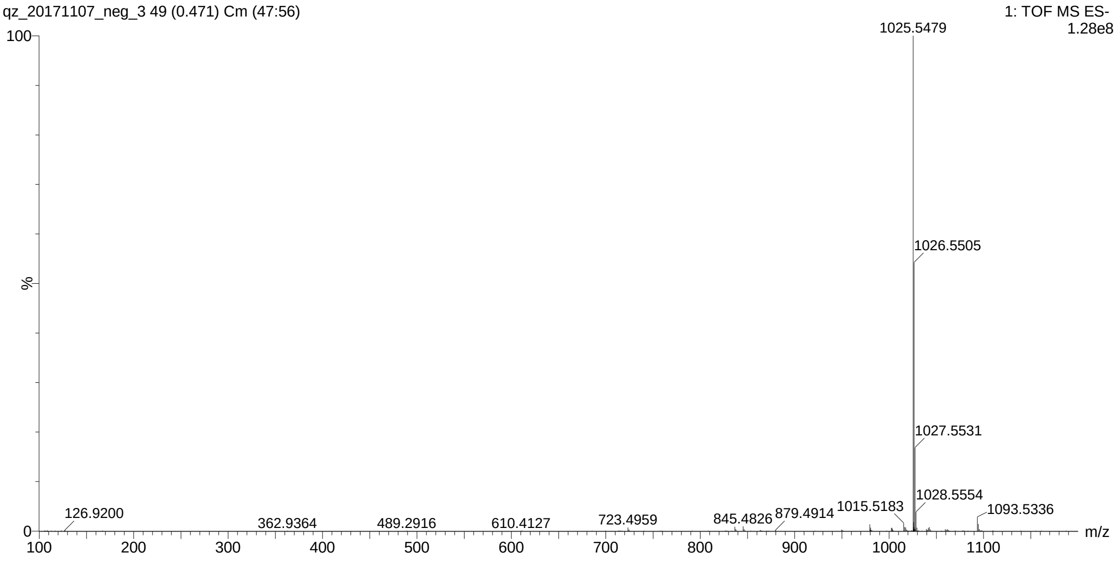

Figure S5-5. High resolution electrospray ionization mass spectroscopy (HRESIMS) spectrum of ginsenoside-S5 (**5**).

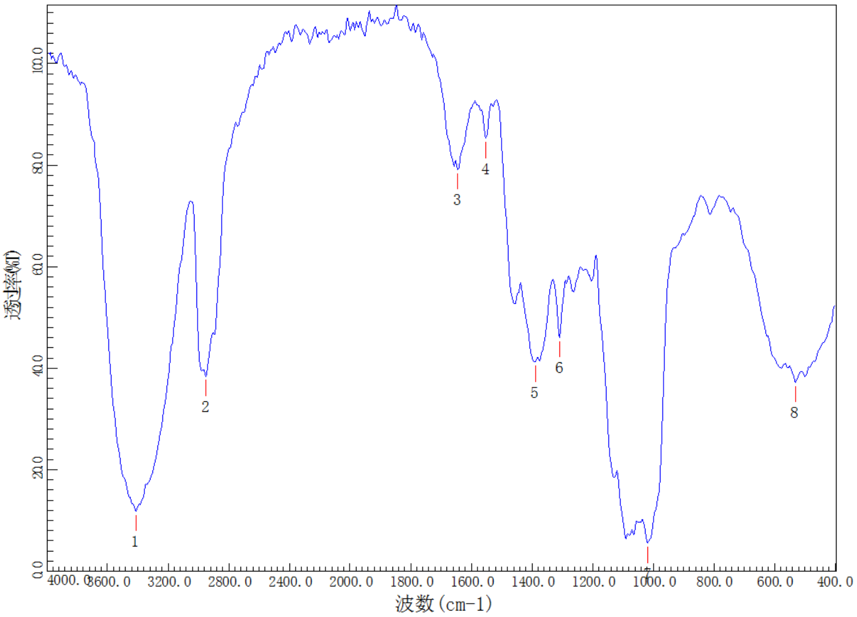

序号	波数(cm-1)	峰值
1	3412.0	11.8
2	2950.0	38.4
3	1644.0	79.1
4	1551.0	85.3
5	1389.0	41.2
6	1308.0	46.0
7	1017.0	5.5
8	531.0	37.2

Figure S5-6. Infrared (IR) spectrum of ginsenoside-S5 (**5**).
